# Twenty‐First‐Century Environmental Change Decreases Habitat Overlap of Antarctic Toothfish ( *Dissostichus mawsoni* ) and Its Prey

**DOI:** 10.1111/gcb.70063

**Published:** 2025-02-10

**Authors:** Cara Nissen, Jilda Alicia Caccavo, Anne L. Morée

**Affiliations:** ^1^ Department of Atmospheric and Oceanic Sciences and Institute of Arctic and Alpine Research University of Colorado Boulder Boulder Colorado USA; ^2^ Department of Freshwater and Marine Ecology, Institute for Biodiversity and Ecosystem Dynamics University of Amsterdam Amsterdam the Netherlands; ^3^ Laboratoire Des Sciences du Climat et de l'Environnement, LSCE/IPSL, CEA‐CNRS‐UVSQ Université Paris‐Saclay Gif‐sur‐Yvette France; ^4^ Laboratoire d'Océanographie et du Climat Expérimentations et Approches Numériques, LOCEAN/IPSL, UPMC‐CNRS‐IRD‐MNHN Sorbonne Université Paris France; ^5^ Climate and Environmental Physics, Physics Institute University of Bern Bern Switzerland; ^6^ Oeschger Centre for Climate Change Research University of Bern Bern Switzerland

**Keywords:** aerobic growth index, Antarctic toothfish, climate change, food web, oxygen, Southern Ocean, temperature

## Abstract

Antarctic toothfish are a commercially exploited upper‐level predator in the Southern Ocean. As many of its prey, the ectothermic, water‐breathing Antarctic toothfish is specifically adapted to the temperature and oxygen conditions present in the high‐latitude Southern Ocean. Additionally, the life cycle of Antarctic toothfish depends on sea‐ice dynamics and the transport of individuals by currents between regions with different prey. To assess the impact of 21st‐century climate change on potential interactions of Antarctic toothfish and its prey, we here employ the extended aerobic growth index (AGI), which quantifies the effect of ocean temperature and oxygen levels on the habitat viability of individual species. We quantify changes in predator–prey interactions by a change in viable habitat overlap as obtained with the AGI. As environmental data, we use future projections for four emission scenarios from the model FESOM‐REcoM, which is specifically designed for applications on and near the Antarctic continental shelf. For the two highest‐emission scenarios, we find that warming and deoxygenation in response to climate change cause a subsurface decline of up to 40% in viable habitat overlap of Antarctic toothfish with important prey species, such as Antarctic silverfish and icefish. Acknowledging regional differences, our results demonstrate that warming and deoxygenation alone can significantly perturb predator–prey habitat overlap in the Southern Ocean. Our findings highlight the need for a better quantitative understanding of climate change impacts on Antarctic species to better constrain future ecosystem impacts of climate change.

## Graphical abstract

Antarctic toothfish are a commercially exploited upper‐level predator in the Southern Ocean, but knowledge on how Antarctic toothfish and its prey respond to environmental change remains incomplete. In this study, we employ the extended aerobic growth index to quantify changes in predator–prey viable habitat overlap in response to changes in temperature and oxygen distributions. We find substantial declines in subsurface habitat overlap between Antarctic toothfish and important prey species, highlighting the need for a more comprehensive assessment of climate‐change impacts on Antarctic species.

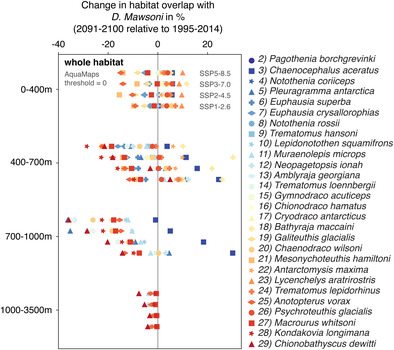

## 1 Introduction

Climate change threatens ecosystem structure and functioning and the exceptional regional biodiversity in the Southern Ocean through ocean warming, sea‐ice decline, changes in circulation, changes in oxygen distributions, and ocean acidification (De Broyer et al. 2014; Douglass et al. 2014; Rogers et al. 2020; Cavanagh et al. 2021; Nissen, Lovenduski, et al. 2024). Upper‐level predators, such as the Antarctic toothfish ( *Dissostichus mawsoni* ), can serve as sentinels of climate change impacts on the wider ecosystem in the Southern Ocean, as they integrate ecological processes from across the food web (Hazen et al. 2019). At the same time, a change in their distribution and hence top‐down pressure can shift ecosystem dynamics. Antarctic toothfish and many of its prey are uniquely adapted to the physical‐biogeochemical conditions of polar waters. Some examples include displaying a low heat tolerance (e.g., fish species within the genus *Trematomus* (Somero and DeVries 1967; Bilyk and DeVries 2011)), having blood with antifreeze proteins (e.g., Antarctic toothfish (DeVries and Cheng 2005)) or without hemoglobin (some icefish species, e.g., the blackfin icefish *Chaenocephalus aceratu* (Ruud 1954; Kim et al. 2019)), or requiring sea ice as a habitat (e.g., Antarctic krill *Euphausia superba* , crystal krill *Euphausia crystallorophias* , Antarctic silverfish *Pleuragramma antarctica* (Corso et al. 2022; Swadling et al. 2023)). Acknowledging the potential for acclimation and further adaptation, these unique characteristics of many Antarctic species imply a pronounced susceptibility of food‐web integrity, that is, food‐web structure and function, to the projected changes in environmental variables, such as temperature, oxygen, or sea‐ice cover across the Southern Ocean (Nissen, Lovenduski, et al. 2024; Pörtner et al. 2019; Roach et al. 2020; Nissen, Timmermann, et al. 2023). Warming and sea‐ice retreat have already altered the distributions of, for example, Antarctic silverfish and Antarctic krill (Rogers et al. 2020; Corso et al. 2022; Atkinson et al. 2019), and substantial changes in high‐latitude temperature and oxygen distributions have been projected for the ongoing century (Nissen, Timmermann, et al. 2023; Nissen et al. 2022). Yet, studies assessing the potential impacts of the projected environmental change on the habitat overlap between Antarctic toothfish and its prey are lacking.

**FIGURE 1:**
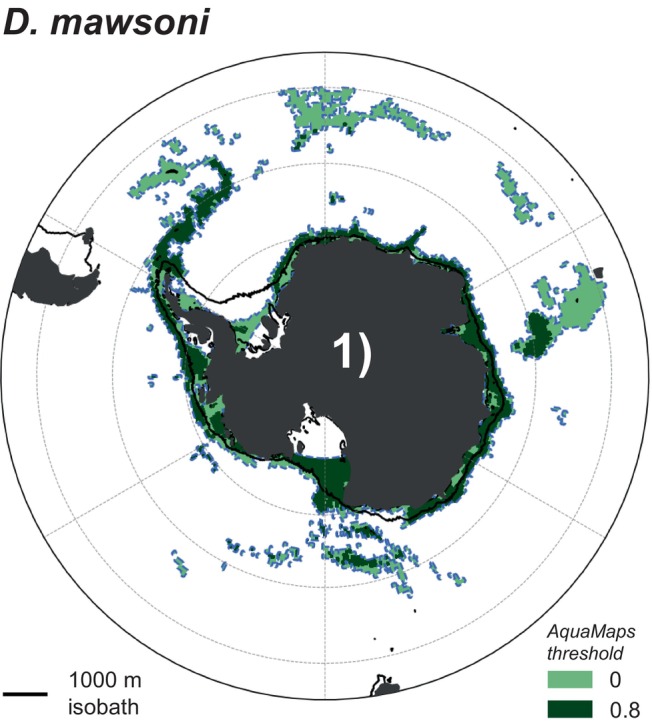
Distribution of the Antarctic toothfish ( *Dissostichus mawsoni* ). The distribution is outlined with the dashed blue line and overlaid with the 1000 m isobath as a solid black line. The different colors correspond to using a probability threshold of species occurrence of zero (light green) and 0.8 (dark green), respectively, in the distribution data from AquaMaps (Kaschner et al. 2019). See Section 2.4 for details on how habitat information was obtained.

The fate of Antarctic toothfish, a notothen, is of particular interest for the future of the Southern Ocean ecosystem: Antarctic toothfish are the largest endemic fish predator in the Southern Ocean, with a trophic level on par with other Southern Ocean top predators such as whales and seals (Jo et al. 2013; Pinkerton and Bradford‐Grieve 2014). Antarctic toothfish reach up to 1–2 m in length over a lifespan that can last decades (Hanchet et al. 2015). Despite its circumpolar distribution (Figure 1), much of our knowledge on the Antarctic toothfish's life history comes from the Ross Sea region (Hanchet et al. 2008; Ashford, Dinniman, and Brooks 2017; Parker et al. 2019). Recognizing gaps in our understanding of its life cycle, the following life history characterized by spawning migrations facilitated through current systems is well supported by previous studies (Hanchet et al. 2008; Ashford, Dinniman, and Brooks 2017; Parker et al. 2019, 2021; Ashford et al. 2012): Spawning occurs over seamounts at the sea‐ice edge in the austral winter (Parker et al. 2019). Eggs hatch after floating to the surface, where larvae develop under sea ice, in an environment with an abundance of prey sources (Parker et al. 2021). In the Ross Sea, developing larvae and spent adults travel with the Ross Gyre toward the continental shelf (Ashford et al. 2012), where fish gain condition feeding on nutrient‐rich prey species such as Antarctic silverfish (*Pleuragramma antarctica*) (La Mesa, Eastman, and Vacchi 2004). Maturing and reconditioned adults are then transported away from the shelf and slope, back toward offshore spawning grounds, where the reproductive cycle begins again (Hanchet et al. 2008).

Antarctic toothfish contribute to the most lucrative fishery in the Southern Ocean, with global exports valued at over 150 million USD in 2021 (CCAMLR Secretariat 2022). The fishery is managed by the Commission for the Conservation of Antarctic Marine Living Resources (CCAMLR), which implements catch limits to ensure biomass remains sufficient to maintain sustainable recruitment and ecosystem maintenance (CCAMLR 1980). This management strategy, however, fails to consider uncertainty due to climate change (Brooks et al. 2018). Results from efforts to quantify potential climate change impacts on Antarctic toothfish distributions remain mixed. Species distribution models have predicted contractions in habitat ranges of Antarctic toothfish, though such efforts did not consider variable climate change impacts throughout the water column (Cheung, Lam, and Pauly 2008; Cheung, Watson, and Pauly 2013). Food‐web modeling suggested a shift toward either smaller phytoplankton species or more gelatinous zooplankton in the ecosystem and a subsequent shift toward energy pathways through fish, but spatially varying environmental change was not explicitly resolved in these studies (Trebilco, Melbourne‐Thomas, and Constable 2020). While such a shift in energy pathways would promote toothfish productivity, increases in illegal, unreported, and unregulated fishing would reverse such gains (Trebilco, Melbourne‐Thomas, and Constable 2020).

The response of Antarctic toothfish to a changing Southern Ocean is intrinsically linked to how its prey respond to environmental change. An opportunistic feeder, the toothfish diet varies across their lifespan and regional habitats, but has been largely shown to include an array of fish and squid species, in particular *Macrourus* spp., icefish, and notothens (Fenaughty, Stevens, and Hanchet 2003; Petrov and Tatarnikov 2011; Roberts, Xavier, and Agnew 2011; Stevens et al. 2014; Yoon et al. 2017; Seong et al. 2021; Lee et al. 2022). While warming generally increases the metabolic demand for oxygen of marine organisms (Pörtner and Knust 2007; Deutsch et al. 2015; Clarke et al. 2021), evidence for climate change impacts on toothfish prey species are mixed (Caccavo et al. 2021). Poleward expansions of subantarctic distributions of *Macrourus* spp. are predicted to impinge on existing *Macrourus* spp. distributions inhabiting high‐latitude waters (Freer et al. 2020). Icefish, with distributions in the Weddell Sea and subantarctic waters of the Atlantic and Indian Ocean sectors, have been shown to have reduced thermal tolerance compared to other Antarctic fish, related to both increased susceptibility to oxidative stress (Mueller et al. 2012), as well as lower aerobic metabolic capacity (O'Brien et al. 2018). This, combined with greater parasitic loads compared with other species (Santoro et al. 2013), renders icefish particularly vulnerable to negative impacts from climate change (Caccavo et al. 2021).

**FIGURE 2:**
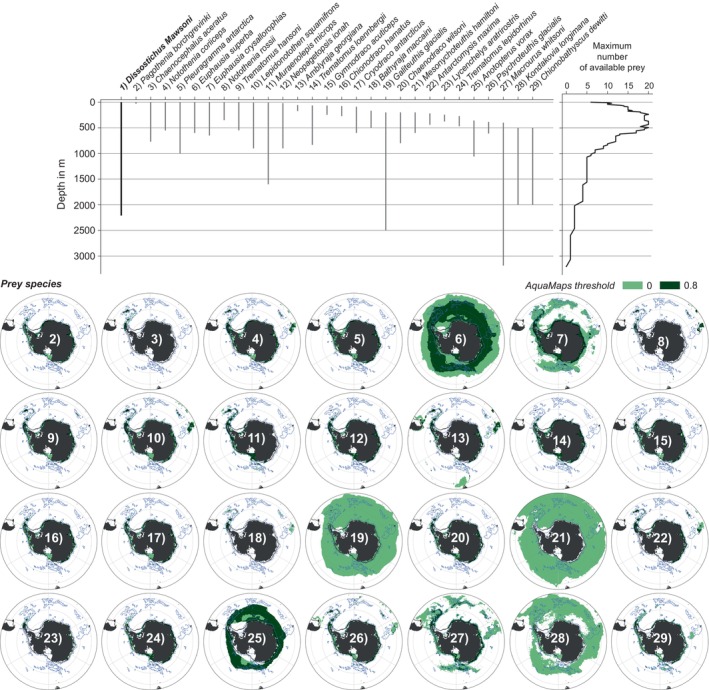
Vertical extent of each species' habitat, including an overview of the maximum number of prey species available at each model depth level. Maps display the species' distributions of the 28 prey of the Antarctic toothfish in filled green contours (numbered 2–29). The different colors correspond to using a probability threshold of species occurrence of zero (light green) and 0.8 (dark green), respectively, in the distribution data from AquaMaps (Kaschner et al. 2019). All distributions are overlaid with the outline of the Antarctic toothfish habitat in dashed blue and the 1000 m isobath as a solid black line. See Sections 2.3 and 2.4 for details on how the list of prey species was compiled and how habitat information was obtained.

Notothens comprise fish from the family Nototheniidae, including important toothfish prey species from the genera *Pleuragramma*, *Trematomus*, *Pagothenia*, *Lepidonotothen*, and *Notothenia* (DeWitt, Heemstra, and Gon 1990). *Pleuragramma antarctica*, or Antarctic silverfish, has a circumpolar distribution on the Antarctic continental shelf (Figure 2) and risks negative consequences from climate change due to two reasons (Corso et al. 2022). First, their early‐life dependence on the platelet ice ecosystem beneath coastal fast ice (Vacchi et al. 2012) and second, their reliance on hydrography to complete life history migrations from coastal areas to the continental slope and back (Ashford, Dinniman, and Brooks 2017). In contrast to *Pleuragramma*'s sensitivity to changes in sea‐ice dynamics and ocean circulation, climate change impacts on other genera were mainly reported for individual species in response to local ocean warming. A lack of heat shock proteins in *Trematomus Bernacchii* Hofmann et al. (2000 ) may reduce *Trematomus* species' ability to acclimate to environmental stress, which could contribute to the restrictions in distributions predicted for *Trematomus* species in East Antarctica under future climate change scenarios (Zhu et al. 2023). Indeed, *Trematomus* species were only partially able to compensate for their physiological response to increased temperatures in an experimental paradigm, as measured by oxygen consumption rates and aerobic capacity. This is in contrast to *Pagothenia borchgrevinki* , which showed near complete physiological compensation to temperature stress (Enzor, Hunter, and Place 2017). *P. borchgrevinki* demonstrated a less robust physiological stress response when exposed to both increasing temperatures and decreasing pH levels, and when acclimation was measured over longer time scales, highlighting the risks of long‐term exposure compounded by multiple concurrent stressors (Huth and Place 2016). Furthermore, a near lack of transcriptional response to heat stress in *P. borchgrevinki* may indicate an inability to physiologically respond to increasing temperatures (Bilyk, Vargas‐Chacoff, and Cheng 2018). While subantarctic distributions of *Lepidonotothen squamifrons* have shown greater physiological plasticity to thermal stress in laboratory settings than *Notothenia rossii* , which inhabits higher‐latitude ecosystems (Strobel et al. 2013), recent evidence for *Lepidonotothen* spawning grounds on the continental shelf supports the existence of distributions in cooler, stabler parts of the Southern Ocean that may be more vulnerable than their subantarctic counterparts (Guo et al. 2023). Species of the genus *Notothenia*, some of which are recovering from years of overexploitation, for example, *N. rossii* Kock and Jones (2005); Barrera‐Oro, Marschoff, and Ainley (2017), showed greater capacity to compensate physiologically to temperature stress (Kandalski et al. 2019; O'Brien et al. 2022), though such compensatory responses may result in underlying cellular and DNA damage Zafalon‐Silva et al. (2017). Finally, species modeling under future climate change scenarios revealed reduced connectivity between populations of *N. rossii* , resulting in isolation that can further exacerbate vulnerability to environmental change Young et al. (2018).

Thus, studies in laboratory settings, modeling distributions, and ecological sampling all point to vulnerabilities among toothfish prey species to climate change. Acknowledging that environmental change could drive each prey to find new suitable habitats, which may or may not overlap with future Antarctic toothfish distributions, highlights the need to understand how prey responses to environmental change may ultimately impact distributions of Antarctic toothfish in a changing Southern Ocean. For continued sustainable fisheries management by CCAMLR, a more detailed quantification of potential climate change impacts on Antarctic toothfish populations across the Southern Ocean is therefore urgently needed.

Warming and deoxygenation are two of the key indicators of the oceanic imprint of 21st‐century climate change (Kwiatkowski et al. 2020). As temperature and oxygen shape marine species distributions (Deutsch et al. 2015; Deutsch, Penn, and Seibel 2020), projections of marine temperature and oxygen provide an opportunity to understand the response of marine species distributions to climate change. Over the past decade, several indices have been developed to quantify the influence of changes in temperature and oxygen supply on marine species distributions, using output from relatively coarse‐resolution climate models as environmental input data (Deutsch et al. 2015; Clarke et al. 2021; Penn et al. 2018). Among all indices, the extended aerobic growth index (AGI) (Clarke et al. 2021; Morée et al. 2023) has a relatively low demand for species‐specific parameters while maintaining agreement with other indices such as the metabolic index (Deutsch et al. 2015). Here we apply AGI to quantify the fraction of contemporary habitat volume that can sustain a viable population of a species, with respect to the influence from temperature and oxygen (Section 2.1). We use temperature and oxygen fields from 21st‐century projections run on a model grid with elevated resolution on and near the Antarctic continental shelf (highest resolution < 5 km; see, for example, ref (Nissen, Lovenduski, et al. 2024; Nissen et al. 2022)), making it an ideal tool for the assessment of changes in viable habitat of Antarctic species (Section 2.2). We apply the AGI for the first time to Southern Ocean species across multiple trophic levels to assess climate change impacts on interactions between an upper‐level predator (Antarctic toothfish) and its most important prey species (Sections 2.3 and 2.4). Our approach therefore facilitates the quantification of how temperature and oxygen changes impact viable habitat overlap between the Antarctic toothfish and its prey.

## 2 Materials and Methods

### 2.1 Calculation of the Aerobic Growth Index and Viable Habitat Overlap

To assess what fraction of contemporary habitat volume is available to sustain a viable population of a particular species, we calculate the extended aerobic growth index (AGI) (Clarke et al. 2021; Morée et al. 2023). AGI assesses habitat viability with respect to in situ temperature *T* (K) and partial pressure of oxygen (*pO*_*2*_ in mbar) and is calculated as the ratio between environmental *pO*_*2*_ supply and species‐specific *pO*_*2*_ demand. For each species *i*, we calculate AGI_i_ at each model grid cell within a species' habitat as

(1) $${\mathrm{AGI}}_{i}=\frac{{\mathrm{pO}}_{2}^{\text{supply}}}{{\mathrm{pO}}_{2,i}^{\text{demand}}}=\frac{{\mathrm{pO}}_{2}^{\text{supply}}}{{\mathrm{pO}}_{2,i}^{\mathrm{thr}}\cdot {\frac{1}{3}}^{1-d}\cdot \exp\left(\frac{j_{2}-j_{1}}{T_{i}^{\text{pref}}}-\frac{j_{2}-j_{1}}{T}\right)},$$

where the temperature dependence is represented by the variables *j*_*1*_ (the anabolism activation energy divided by the Boltzmann constant, 4500 K), *j*_*2*_ (the catabolism activation energy divided by the Boltzmann constant, 8000 K), and *d* (the metabolic scaling coefficient, 0.7). The species‐specific threshold of *pO*_*2*_ (${\mathrm{pO}}_{2,i}^{\mathrm{thr}}$) and preferred temperature ($T_{i}^{\text{pref}}$) are calculated as the volume‐weighted 10th and 50th percentile over monthly climatological in‐habitat *pO*_*2*_ and *T* (1995–2014), respectively (Table 1). We then calculate the critical threshold of ${\mathrm{AGI}}_{i}$ (${\mathrm{AGI}}_{i}^{\text{crit}}$) as the 10th percentile of volume‐weighted monthly climatological in‐habitat AGI (1995–2014; Table 1). When ${\mathrm{AGI}}_{i}$ is above ${\mathrm{AGI}}_{i}^{\text{crit}}$, that part of the contemporary habitat can sustain a viable population of species *i*. Thus, the total viable habitat volume Ω in km^3^ of species *i* can be obtained by summing up the volume V of all model grid cells n with ${\mathrm{AGI}}_{i}$ > ${\mathrm{AGI}}_{i}^{\text{crit}}$:

(2) $$\Omega_{i}=\sum_{n\in \left\{{\mathrm{AGI}}_{i}>{\mathrm{AGI}}_{i}^{\text{crit}}\right\}}V_{n}.$$

**TABLE 1:** Overview of key characteristics of all species assessed in this study. Displayed are the minimum and maximum depth of occurrence (see references below the table), the preferred temperature ($T^{\text{pref}}$), the threshold in partial pressure of oxygen (${\mathrm{pO}}_{2}^{\mathrm{thr}}$), and the critical aerobic growth index (${\mathrm{AGI}}^{\text{crit}}$) within the habitat of each species. We use monthly climatological fields of in situ temperature and ${\mathrm{pO}}_{2}$ to compute $T^{\text{pref}}$, ${\mathrm{pO}}_{2}^{\mathrm{thr}}$, and ${\mathrm{AGI}}^{\text{crit}}$ (see Section 2.1). The horizontal and vertical extent of each species' habitat is shown in Figure 2. Note that no common names are available below family level for *Chaenodraco wilsoni* and *Chionobathyscus dewitti* , thus the same common name, crocodile icefish, is indicated.

# in Figures 1 and 2	Species name	Common name	Min. depth in m	Max. depth in m	References for depth levels	T^*pref*^ in °C	*pO*_*2*_^*thr*^ in mbar	AGI_*crit*_
1	*Dissostichus mawsoni*	Antarctic toothfish	0	2210	a	+0.06 (−0.18)	120.2 (120.4)	1.26 (1.35)
2	*Pagothenia borchgrevinki*	Bald rockcod	0	30	b	−1.53 (−1.51)	160.5 (166.3)	1.40 (1.40)
3	*Chaenocephalus aceratus*	Blackfin icefish	0	770	c	+0.22 (+0.82)	118.7 (121.5)	1.25 (1.24)
4	*Notothenia coriiceps*	Black rockcod	0	550	d	−0.73 (−0.66)	127.1 (125.4)	1.22 (1.13)
5	*Pleuragramma antarctica*	Antarctic silverfish	0	1000	e	−0.77 (−0.72)	125.2 (123.8)	1.34 (1.34)
6	*Euphausia superba*	Antarctic krill	0	600	f	+1.80 (+0.88)	117.7 (116.1)	1.34 (1.32)
7	*Euphausia crystallorophias*	Ice krill	0	650	b	+0.55 (−0.78)	116.6 (121.7)	1.28 (1.32)
8	*Notothenia rossii*	Marbled rockcod	5	350	e	+3.02 (+3.68)	129.5 (132.0)	1.40 (1.38)
9	*Trematomus hansoni*	Striped rockcod	6	549	e	−0.87 (−0.82)	127.2 (124.8)	1.30 (1.24)
10	*Lepidonotothen squamifrons*	Grey rockcod	10	900	e	−0.26 (+0.55)	122.8 (122.3)	1.17 (1.20)
11	*Muraenolepis microps*	Smalleye moray cod	10	1600	e	−0.12 (−0.21)	120.6 (122.6)	1.31 (1.29)
12	*Neopagetopsis ionah*	Jonah's icefish	20	900	e	−0.68 (−0.70)	123.2 (124.4)	1.34 (1.34)
13	*Amblyraja georgiana*	Antarctic starry skate	57	173	b	+1.88 (−0.02)	141.6 (141.0)	1.48 (1.37)
14	*Trematomus loennbergii*	Scaly rockcod	65	832	b	−0.71 (−0.85)	123.2 (125.8)	1.34 (1.33)
15	*Gymnodraco acuticeps*	Ploughfish	66	247	b	−1.06 (−1.04)	139.0 (136.3)	1.33 (1.32)
16	*Chionodraco hamatus*	Crocodile icefish	76	271	b	−1.00 (−0.99)	135.0 (129.2)	1.32 (1.31)
17	*Cryodraco antarcticus*	Long‐fingered icefish	90	600	e	−0.81 (−0.76)	126.3 (122.0)	1.33 (1.32)
18	*Bathyraja maccaini*	McCain's skate	167	500	d	+3.07 (0.02)	120.9 (117.3)	1.35 (1.33)
19	*Galiteuthis glacialis*	Glass squid	200	2500	g	+9.91 (+9.91)	121.3 (121.3)	1.92 (1.92)
20	*Chaenodraco wilsoni*	Spiny icefish	200	800	e	−0.53 (−0.54)	121.3 (120.5)	1.35 (1.35)
21	*Mesonychoteuthis hamiltoni*	Colossal squid	200	600	h	+2.80 (+2.80)	117.0 (117.0)	1.40 (1.40)
22	*Antarctomysis maxima*	Opossum shrimp	220	440	b	−0.25 (−0.39)	123.2 (122.8)	1.18 (1.19)
23	*Lycenchelys aratrirostris*	Lycodine eelpout	244	376	b	−0.76 (−0.79)	119.9 (127.39)	1.31 (1.33)
24	*Trematomus lepidorhinus*	Slender scalyhead	272	468	b	−0.65 (−0.90)	122.3 (129.0)	1.34 (1.33)
25	*Anotopterus vorax*	Southern Ocean daggertooth	358	1059	b	+0.25 (+0.27)	117.8 (117.7)	1.32 (1.32)
26	*Psychroteuthis glacialis*	Glacial squid	385	610	b	+0.03 (−0.08)	119.7 (118.3)	1.19 (1.22)
27	*Macrourus whitsoni*	Whitson's grenadier	400	3185	e	+0.06 (−0.15)	122.1 (121.7)	1.24 (1.33)
28	*Kondakovia longimana*	Oceanic squid	500	2000	i	+0.64 (+0.64)	121.5 (121.5)	1.27 (1.27)
29	*Chionobathyscus dewitti*	Crocodile icefish	500	2000	e	−0.13 (−0.25)	120.6 (125.2)	1.36 (1.37)

*Note:* a ref (Hanchet et al. 2008); b ref (Kaschner et al. 2019); c (Reid et al. 2007); d https://fishesofaustralia.net.au (last access: March 9, 2024); e https://www.fishbase.se (last access: March 9, 2024); f https://www.marinespecies.org (last access: March 9, 2024); g ref (Piatkowski and Hagen 1994); h https://www.fao.org/3/ac479e/ac479e00.htm (last access: March 9, 2024); i ref (Lu and Williams 1994).

We compute the overlap Φ in km^3^ in viable habitat of the Antarctic toothfish and each prey species *j* by summing up the volume of model grid cells, where AGI_*D.mawsoni*_ > $\;{\mathrm{AGI}}_{D.\;\text{mawsoni}}^{\text{crit}}$ and ${\mathrm{AGI}}_{j}{>\mathrm{AGI}}_{j}^{\text{crit}}$, that is, by assessing where $\Omega_{D.\text{mawsoni}}$ intersects $\Omega_{j}$:

(3) $$\Phi_{j}=\Omega_{D.\text{mawsoni}}\cap \Omega_{j}.$$

The extended version of AGI used here (Morée et al. 2023) is less sensitive to ${\mathrm{pO}}_{2}$ and *T* change than the original AGI (Clarke et al. 2021) because it relies on monthly 3D habitat data for the calculation of ${\mathrm{pO}}_{2,i}^{\mathrm{thr}}$, $T_{i}^{\text{pref}}$, and ${\mathrm{AGI}}_{i}^{\text{crit}}$ instead of surface ocean or sea floor data only. Seasonal variability and the strong vertical gradients of ${\mathrm{pO}}_{2}$ and *T* are therefore included in the calculation of ${\mathrm{pO}}_{2,i}^{\mathrm{thr}}$, $T_{i}^{\text{pref}}$, and ${\mathrm{AGI}}_{i}^{\text{crit}}$.

To assess future changes, we first calculate the relative future change in AGI (${\mathrm{AGI}}_{\mathrm{rel}}$; in %) with

(4) $${\mathrm{AGI}}_{\mathrm{rel}}=100\cdot \left(\frac{{\mathrm{AGI}}^{\text{future}}}{{\mathrm{AGI}}^{1995-2014}}-1\right).$$

We note that, by definition (see also Equation 1), ${\mathrm{AGI}}_{\mathrm{rel}}$ is species independent and reflects changes in temperature and oxygen distributions for a given region. It is useful as a measure of the direction and magnitude of change of habitat viability across species. Next, we assess species‐specific future changes in viable habitat Ω (Equation 2) and habitat overlap Φ (Equation 3) for the Antarctic toothfish and each of its prey. For each species, we quantify the change in 3D in‐habitat volume of waters with ${\mathrm{AGI}}_{i}>{\mathrm{AGI}}_{i}^{\text{crit}}$ between 1995 and 2014 and a future time slice, using drift‐corrected annual mean fields of in situ temperature and ${\mathrm{pO}}_{2}$ (see Section 2.2). Besides reporting results for the whole Antarctic toothfish habitat and all prey species, we provide additional detail for selected parts of the analysis. Motivated by many prey species exclusively inhabiting Antarctic coastal waters (Figure 2), we separately quantify the change in habitat overlap Φ for the Antarctic continental shelf, which we here define as the area south of the 1000 m isobath of the model grid. Further, to highlight regional differences in changes in habitat overlap Φ, we separately report hotspots of prey loss for the CCAMLR Convention Areas 48 (Weddell Sea), 58 (East Antarctica), and 88 (Ross Sea, Amundsen Sea, Bellingshausen Sea) (CCAMLR Convention Areas 2017). Throughout the manuscript, we report results as a function of depth, acknowledging that the Antarctic toothfish inhabits different depth levels at different life cycle stages (Hanchet et al. 2008; Ashford, Dinniman, and Brooks 2017; Parker et al. 2019). For the habitat overlap Φ, we synthesize the results into distinct depth intervals (0–400 m, 400–700 m, 700–1000 m, 1000–3500 m), whose choice was mainly guided by the projected change in the vertical distribution of oxygen (Figure 4b).

By using annual mean fields in the assessment of future changes (as opposed to monthly means), the results presented hereafter are on the conservative side; changes in viable habitat and habitat overlap could be larger if monthly fields were used. We note that the sensitivity of our results to the chosen time period appears to be small (as an example, compare 2081–2100 and 2098–2100 to 2091–2100 in Figure 8b), and we will focus the presentation of the results to the period 2091–2100. Last, we attribute the projected changes in AGI to projected changes in temperature (oxygen) by keeping ${\mathrm{pO}}_{2}$ (in situ temperature) at 1995–2014 levels in the AGI computation. Since ${\mathrm{pO}}_{2}$ depends on both temperature and oxygen (see above), 2091–2100 ${\mathrm{pO}}_{2}$ fields are recalculated for the attribution analysis by using drift‐corrected 2091–2100 oxygen concentrations and present‐day climatological in situ temperatures (1995–2014).

### 2.2 Temperature and Oxygen Data: Description of FESOM‐REcoM Model Simulations

As environmental data, we use the global ocean‐sea ice model FESOM version 1.4 (referred to as “FESOM” hereafter) (Wang et al. 2014; Danilov et al. 2015), which includes an explicit representation of ice‐shelf cavities (Timmermann, Wang, and Hellmer 2012). FESOM is coupled to the biogeochemical model REcoM version 2 (“REcoM” hereafter) (Hauck et al. 2013; Karakuş et al. 2021), which resolves the cycling of carbon, nitrogen, silicon, iron, and oxygen. The version of REcoM used here includes two phytoplankton groups (diatoms and mixed nanophytoplankton) and two zooplankton groups (microzooplankton and Antarctic krill) (Karakuş et al. 2021).

For this study, all FESOM‐REcoM simulations are run on an unstructured, multi‐resolution mesh with eddy‐permitting resolution on the high‐latitude Antarctic continental shelf and coarser resolution in the open ocean; the horizontal resolution ranges from < 5 to 150 km across the Southern Ocean (see Figure S8 in Nissen, Lovenduski, et al. 2024). The mesh has 99 *z*‐level in the vertical, which decrease in resolution with depth (78 of all depth levels are above 2000 m). All model experiments are forced with three‐hourly atmospheric output from the AWI Climate Model, which contributed to the sixth phase of the “Coupled Model Intercomparison Project” (Semmler et al. 2020). Model tracers are initialized on January 1, 1950 with output from FESOM within the AWI Climate Model (all physical model tracers) (Semmler et al. 2020) and REcoM in simulations for the “REgional Carbon Cycle Assessment and Processes” project (RECCAP; all biogeochemical tracers) (DeVries et al. 2023; Hauck et al. 2023). After a historical simulation from 1950 to 2014, FESOM‐REcoM is run from 2015 to 2100 for four Shared Socioeconomic Pathways (SSP scenarios): SSP1‐2.6, SSP2‐4.5, SSP3‐7.0, and SSP5‐8.5 (sorted from low to high greenhouse gas emissions as well as high to low mitigation efforts). In addition, a control simulation is performed, in which all atmospheric forcing variables are held constant at conditions of the year 1950 (atmospheric CO_2_) or 1955 (all other variables such as wind, air temperature, or precipitation) (Nissen et al. 2022). Note that these model simulations are evaluated, analyzed, and described in more detail in Nissen, Lovenduski, et al. (2024); Nissen, Timmermann, et al. (2023); Nissen et al. (2022).

For this study, we use monthly FESOM‐REcoM output of potential temperature, practical salinity, and oxygen concentration from 1995 to 2014 from the historical and control simulations and from 2081 to 2100 from the control simulation and all SSP scenarios. All model output is first linearly interpolated onto a regular mesh with 0.125° × 0.25° (latitude × longitude) horizontal resolution. In the vertical, we reduce the output to 0–3185 m (top 88 depth levels in the model), as none of the prey species has a habitat below this depth level (see Figure 2, Table 1, and Section 2.4). In line with the northerly extent of the toothfish habitat (Figure 1), we further reduce the model output to south of 45°S. To isolate the climate change signal in our analysis, we correct the future fields of temperature and oxygen for model drift by subtracting the change in the respective variable in the control simulation between future conditions and 1995–2014. To assess the sensitivity of our results to the chosen future time period, three different future time periods are used: 2091–2100, 2081–2100, and 2098–2100 (see also Section 2.1). As the calculation of the AGI relies on in situ temperature and ${\mathrm{pO}}_{2}$ (see Section 2.1), we then first compute in situ temperature and density fields from the modeled potential temperature and practical salinity using python's seawater library. Oxygen concentrations are converted from model units (mmol m^−3^) to mol kg^−1^ using the in situ density fields, and *p*O_2_ is computed from in situ temperature and oxygen concentrations in mol kg^−1^ following Appendix B in Morée et al. (2023). All calculated model fields of in situ temperature and *p*O_2_ used in this study are available on Zenodo (Nissen 2024).

#### 2.2.1 Model Evaluation

**FIGURE 3:**
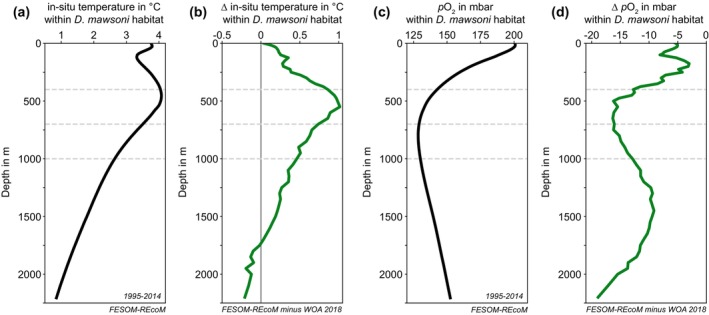
(a, b) Vertical profiles of in situ temperature in °C within the habitat of the Antarctic toothfish ( *Dissostichus mawsoni* ) (a) in FESOM‐REcoM for 1995–2014 and (b) as the FESOM‐REcoM bias from WOA18 climatological observation data WOA 2018 (Locarnini et al. 2018). For the comparison with WOA data, FESOM‐REcoM data are linearly interpolated in the vertical to the depth levels of WOA data before computing the bias within the toothfish habitat. (c, d) same as (a, b), but for partial pressure of oxygen (*pO*_*2*_) in mbar (Garcia et al. 2018; Zweng et al. 2018). Horizontal lines are shown at depths of 400 m, 700 m, and 1000 m, illustrating the depth intervals chosen for the results shown in Figures 6 and 7, and S1–S4.

Within the Antarctic toothfish habitat, both in situ temperature and ${\mathrm{pO}}_{2}$ are highest in the upper ocean above 500 m and lower below that depth (Figure 3a,c). In order to assess model bias, we compare with data from the World Ocean Atlas (WOA2018) (Locarnini et al. 2018; Garcia et al. 2018; Zweng et al. 2018), acknowledging that WOA2018 is associated with uncertainties in the Southern Ocean due to the limited spatial and temporal observational coverage. Over the historical period 1995–2014, simulated in situ temperatures in FESOM‐REcoM are higher within the top 1750 m of the Antarctic toothfish habitat than WOA2018 data (Figure 3b) (Locarnini et al. 2018). Simultaneously, simulated ${\mathrm{pO}}_{2}$ is lower than in WOA2018 throughout the water column (Figure 3d; the calculation of ${\mathrm{pO}}_{2}$ in WOA2018 follows the one described for the model output in Section 2.1) (Garcia et al. 2018; Zweng et al. 2018). For both temperature and oxygen, the largest model bias is located near 500 m depth, where the discrepancy between FESOM‐REcoM and WOA2018 averages to +0.96°C (Figure 3b) and −17.6 mbar (Figure 3d), respectively. The positive (negative) bias in temperature (oxygen) implies that the preferred temperature $T^{\text{pref}}$ (threshold in partial pressure of oxygen ${\mathrm{pO}}_{2}$) given in Table 1 is also biased high (low). We note that model biases in temperature and oxygen are larger for some locations outside of the Antarctic toothfish habitat (see Figure S1), which affect the calculated preferred temperature and the threshold in ${\mathrm{pO}}_{2}$ of any prey species whose habitat overlaps with those regions. However, given that these model biases likely affect the historical and future time slices similarly, we assume that the effect of present‐day model biases on projected changes in temperature and oxygen and hence relative changes in AGI is negligible for the subsequent analysis.

### 2.3 Compilation of Most Important Prey for the Antarctic Toothfish

The list of prey species included in the analysis is the result of an in‐depth literature search combined with an assessment of prey importance. All prey identified in the literature review (see Tables S1 and S2 and refs (Jo et al. 2013; Fenaughty, Stevens, and Hanchet 2003; Petrov and Tatarnikov 2011; Roberts, Xavier, and Agnew 2011; Yoon et al. 2017; Seong et al. 2021; Eastman 1985; Near et al. 2003; Stevens 2004; Stevens 2006; Park et al. 2015; Petrov and Gordeev 2015)) were checked against the SCAR Diet and Energetics database (Scientific Committee on Antarctic Research 2023). Of the 72 prey species identified in the literature search, 67 were also identified in the SCAR database. One species ( *Aptenodytes forsteri* , Emperor penguin) was identified in the SCAR database that was not present among the 72 species we identified. However, it was not possible to corroborate this database finding with referenced literature, and thus the species was not included in the final prey database. The list of 72 prey species to be included in the analysis was further reduced by taking into account the abundance and frequency of prey species identified in diet studies, and the extent to which prey species distributions were restricted to the Southern Ocean (Tables S1 and S2). This resulted in a final 28 prey species that were included in the AGI analysis (Figure 2 and Table 1).

### 2.4 Species Distributions

For the calculation of viable habitat overlap between the Antarctic toothfish and its prey, we used three‐dimensional distribution data for all species (Figures 1 and 2). First, two‐dimensional (horizontal) distribution data were taken from AquaMaps (Kaschner et al. 2019) except for the squid *Galiteuthis glacialis* , *Kondakovia longimana* , and *Mesonychoteuthis hamiltoni* , which are taken from Xavier et al. (2016) and Raymond et al. (2015). The 0.1° × 0.1° gridded distributions from Xavier et al. (2016) and Raymond et al. (2015) are derived from a combination of observational occurrence data and modeling of habitat suitability. The squid species are considered to have their habitat anywhere where habitat suitability exceeds the habitat suitability thresholds of 0.228 for *Galiteuthis glacialis* , 0.281 for *Kondakovia longimana* , and 0.121 for *Mesonychoteuthis hamiltoni* (threshold values as in Xavier et al. (2016), personal comm.).

The native distribution maps from AquaMaps (Kaschner et al. 2019) are 0.5° × 0.5° gridded data that consist of species occurrence probabilities. For our study, we considered a species to have its habitat anywhere where the probability is larger than zero. Since the northern regions of the resulting Antarctic toothfish habitat are associated with relatively warmer waters and therefore impact the critical AGI used as the basis for our analysis (see Section 2.1), we additionally repeated our calculations with a species occurrence probability threshold of 0.8 (Table 1), which results in a smaller overall habitat of Antarctic toothfish and its prey (Figures 1 and 2). In Section 3, we report results using both thresholds for selected parts of the analysis. We refer to Hodapp et al. (2023) for more details on the AquaMaps methodology. All AquaMaps data were reviewed by AquaMaps prior to use in this study. All two‐dimensional distribution data were regridded to the model grid using largest area fraction regridding (Schulzweida 2022) before further analysis, thereby excluding any occurrence north of the model limit at 45° S. In doing so, we excluded a small fraction of the habitat of *Amblyraja georgiana* (presence up to 43.25° S) and the squid *Kondakovia longimana* and *Mesonychoteuthis hamiltoni* (presence up to 40° S), which we assume to have a negligible effect on our AGI calculations (see Section 2.1 and Table 1). Second, we extend the two‐dimensional distribution data over the full species‐specific depth range between the minimum and maximum depth of occurrence, as shown in Figures 1 and 2 and listed in Table 1, resulting in a three‐dimensional distribution for each species. All species distributions are assumed to represent the distribution of each species during the 1995–2014 time period. Morée, Caccavo, and Nissen (2024) and Nissen et al. provide a collection of all distribution data used for this study, regridded to the model grid.

## 3 Results

### 3.1 Exposure of Antarctic Toothfish and Its Prey to Environmental Change

**FIGURE 4:**
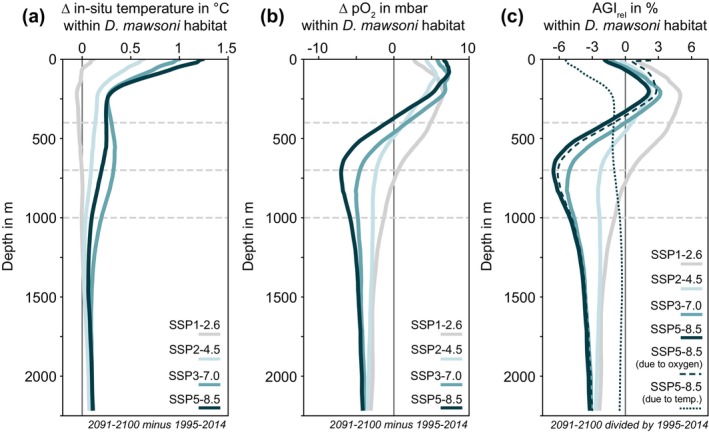
Vertical profiles of the drift‐corrected change in (a) in situ temperature in °C, (b) partial pressure of oxygen (${\mathrm{pO}}_{2}$) in mbar, and (c) the aerobic growth index (AGI_*rel*_; Equation 4) in percent within the habitat of the Antarctic toothfish ( *Dissostichus mawsoni* ) between 2091 and 2100 for the four emission scenarios and 1995–2014. To obtain the habitat, a species occurrence probability threshold of zero was used (light green in Figure 1). In panel (c), the dashed and dotted lines denote the future change due to only oxygen and temperature, respectively (SSP5‐8.5 scenario). Horizontal lines are shown at depths of 400 m, 700 m, and 1000 m, illustrating the depth intervals chosen for the results shown in Figures 6, 7, S1–S4.

By the 2090s, waters within the Antarctic toothfish habitat are projected to undergo warming and a redistribution of oxygen (Figure 4a,b). Wsaters above 1000 m are projected to warm substantially for all but the lowest emission scenario (Figure 4a and Figure S2). For the highest emission scenario, the warming within the Antarctic toothfish habitat amounts to up to 1.24°C at the surface, while this is about 0.62°C for the intermediate scenario SSP2‐4.5 and close to zero for the lowest emission scenario (Figure 4a). Interestingly, the maximum warming below 500 m of 0.34°C is simulated for the second‐highest emission scenario SSP3‐7.0, illustrating the importance of not only atmospheric warming levels but also internal variability for projections of oceanic change. Regardless of the emission scenario, oxygen concentrations are projected to increase in the upper ocean above 500–750 m and decrease below (drift‐corrected; Figure 4b and S3). While the increase in oxygen partial pressure in the top 250 m varies from 5.6 to 7.4 mbar across scenarios (Figure 4b), the average decrease at the subsurface below 500 m exceeds 7 mbar in the highest emission scenario but amounts to only 3 mbar in the lowest emission scenario.

Changes in AGI relative to the reference period 1995–2014 (AGI_*rel*_; Equation 4) capture the direction of change of habitat viability for a given region due to the described warming and deoxygenation. As expected, mean AGI_*rel*_ in the “Antarctic toothfish” habitat is largely negative, indicating reduced habitat viability by 2091–2100 relative to 1995–2014 (Figures 4c, S4, S5). Changes in $O_{2}$ dominate the projected change in AGI_*rel*_ (compare the dashed and solid dark green lines in Figures 4c, S4, S5). By the end of the century, AGI_*rel*_ within the Antarctic toothfish habitat increases by up to 5% in the top 400 m for the lowest emission scenario and declines by up to 6.5% below 400 m for the highest emission scenario (Figure 4c). Within the prey habitats (Figure 2), the top 400 m increase in AGI_*rel*_ amounts to more than 10% for blackfin icefish ( *C. aceratus* ), whereas the decline below 400 m exceeds 10% for some depth levels for Antarctic silverfish ( *P. antarctica* ), Jonah's icefish ( *N. ionah* ), and spiny icefish ( *C. wilsoni* ; see Figure S5). In general, this relative change in AGI affects all species in a given region, but the realized impact depends on species‐specific thresholds (Table 1). Consequently, the change in AGI may impact both the viable habitat of the Antarctic toothfish and the overlap with the viable habitat of its prey when locally decreasing AGI to below AGI_*crit*_.

### 3.2 Projected Environmental Change Drives Decline in Viable Habitat

**FIGURE 5:**
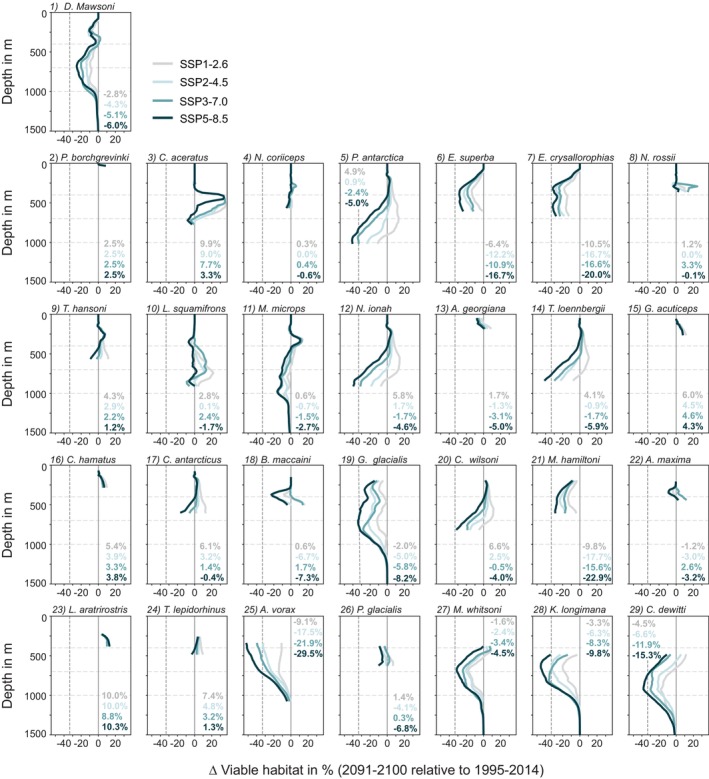
Vertical profiles of the drift‐corrected change in viable habitat (Ω; Equation 2) in % for (1) the Antarctic toothfish ( *Dissostichus mawsoni* ) and (2) 29 each of its prey between 2091 and 2100 for the four emission scenarios and 1995–2014. To obtain the habitat, a species occurrence probability threshold of zero was used (light green in Figures 1 and 2). The sorting of all species is identical to the sorting in Figure 2. The vertical gray lines denote no change in viable habitat (solid) and a one‐third decline (dashed). Horizontal lines are shown at depths of 400 m, 700 m, and 1000 m, illustrating the depth intervals chosen for the results shown in Figures 6 and 7, S1–S4. Numbers in each panel correspond to the vertically averaged change in viable habitat in percent.

In response to the projected environmental change, the majority of species experiences a decline in viable habitat Ω for at least some emission scenarios (Figure 5; Equation 2; to obtain the habitat, the probability threshold of species occurrence is zero). Averaged over their respective 3D habitat, the 2091–2100 loss of the historical viable habitat is largest for Southern Ocean daggertooth ( *A. vorax* ; up to 29.5% in the highest emission scenario, see printed numbers in Figure 5), colossal squid ( *M. hamiltoni* ; up to 22.9%), ice krill ( *E. crystallorophias* ; up to 20.0%), Antarctic krill ( *E. superba* ; up to 16.7%), and crocodile icefish ( *C. dewitti* ; up to 15.3%). Except for crocodile icefish, these species' habitats are restricted to the top ~1000 m and include some open‐ocean areas (see Figure 2), where particularly large changes in temperature and oxygen concentrations may explain the pronounced viable habitat loss for the highest emission scenario (Figures S6,S7). For most species, differences in the averaged viable habitat change across emission scenarios are smaller than 10%. However, scenario differences exceed 10% for the species with the largest projected viable habitat loss ( *A. vorax* , *M. hamiltoni* , *E. superba* , *C. dewitti* ; Figure 5), implying substantial benefits for these species from emission mitigation. Integrated over all species, 18% (46%) of all prey species are projected to experience a decline of 10% (5%) of their historical viable habitat for the high‐emission scenario SSP5‐8.5; the decline amounts to only 4% (14%) of all prey species for the lowest emission scenario SSP1‐2.6.

Projected loss in viable habitat is largest at the subsurface (Figure 5), which is in agreement with the depths of maximum deoxygenation (Figures 4b, S6). While the viable habitat in the top ~250 m remains largely unchanged for most species in response to the compensating effects of higher ${\mathrm{pO}}_{2}$ and higher temperature (Figures 4c, S6, S7), the projected subsurface viable habitat loss is largest between ~500 and 1200 m. For these depth levels, losses in viable habitat are substantial for some species for the highest emission scenario, exceeding one third for Antarctic silverfish ( *P. antarctica* ), Jonah's icefish ( *N. ionah* ), scaly rockcod ( *T. loennbergii* ), glass squid ( *G. glacialis* ), Southern Ocean daggertooth ( *A. vorax* ), Oceanic squid ( *K. longimana* ), and crocodile icefish ( *C. dewitti* ), with many more experiencing a projected viable habitat loss of more than 20% at some depths (e.g., Antarctic toothfish/ *D. mawsoni* ). For these most‐affected species, subsurface viable habitat loss is reduced substantially in the lower emission scenarios (compare light gray and light cyan lines to dark green line in Figure 5), with some species even experiencing an end‐of‐century increase in subsurface viable habitat for the lowest emission scenario SSP1‐2.6 (e.g., Antarctic silverfish/ *P. antarctica* ), emphasizing the potential of climate change mitigation to reduce impact on these species.

### 3.3 Decline in Viable Habitat Creates Hotspots of Prey Loss for the Antarctic Toothfish

**FIGURE 6:**
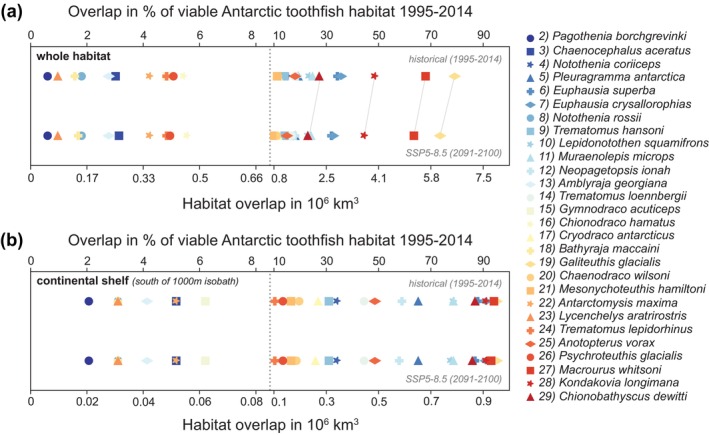
Overlap in drift‐corrected viable habitat (Φ; Equation 3) for the whole water column between the Antarctic toothfish ( *Dissostichus mawsoni* ) and its prey for the historical time period (1995–2014) and for the high‐emission scenario SSP5‐8.5 (2091–2100). The overlap is shown in percent of the viable *D. mawsoni* habitat 1995–2014 (top *x* axis) and in 10^6^ km^3^ (bottom *x* axis) and for (a) the whole *D. mawsoni* habitat and (b) in the fraction of the *D. mawsoni* habitat on the Antarctic continental shelf south of the 1000 m isobath (see Figure 1). Note that the vertical dotted gray lines denotes a change in the scaling of both *x* axes. The thin gray lines highlight the species with the largest absolute decline in viable habitat overlap. To obtain the habitat, a species occurrence probability threshold of zero was used (light green in Figures 1 and 2). The sorting of prey species corresponds to the sorting in Figure 2.

The distinct projected changes in viable habitat (Ω; Equation 2) translate into corresponding changes in viable habitat overlap between the Antarctic toothfish and its prey (Φ; Equation 3). Initially (1995–2014), the magnitude of total viable habitat overlap between the Antarctic toothfish and its prey varies by more than two orders of magnitude (Figure 6; to obtain the habitat, the probability threshold of species occurrence is zero), ranging from 0.05 × 10^6^ km^3^ for bald rockcod ( *P. borchgrevinki* ; 0.6% of the total Antarctic toothfish habitat; dark blue circle in Figure 6a) to 6.6 × 10^6^ km^3^ for glass squid ( *G. glacialis* ; 79%; yellow diamond) for the whole toothfish habitat. Similarly, on the continental shelf, the overlap ranges from 0.02 × 10^6^ km^3^ for bald rockcod (2%) to 0.92 × 10^6^ km^3^ for glass squid (95%; Figure 6b). Unsurprisingly, by the end of the 21st century, prey species with a large present‐day overlap are projected to experience the largest absolute decline in habitat overlap for the highest‐emission scenario: 0.49 × 10^6^ km^3^ for glass squid ( *G. glacialis* ; see thin gray lines in Figure 6a), 0.44 × 10^6^ km^3^ for crocodile icefish ( *C. dewitti* ; red triangle), 0.4 × 10^6^ km^3^ for Whitson's grenadier ( *M. whitsoni* ; red square), and 0.35 × 10^6^ km^3^ for Oceanic squid ( *K. longimana* ; red star). The strongest relative decline in viable habitat overlap is projected for the prey species with largest declines in viable habitat (see Section 3.2), that is, crocodile icefish ( *C. dewitti* ; −19.7%), Southern Ocean daggertooth ( *A. vorax* ; −17.8%; orange diamond), and colossal squid ( *M. hamiltoni* ; −17.1%; orange square). Eight additional prey species with an initial (1995–2014) overlap of > 10% (area to the right of the vertical dotted gray line in Figure 6a) are projected to lose between 5% and 10% in viable habitat overlap for the SSP5‐8.5 scenario ( *K. longimana* 8.8%, *E. crystallorophias* 8.3%, *P. antarctica* 7.8%, *G. glacialis* 7.4%, *M. whitsoni* 7.1%, *E. superba* 6.8%, *T. loennbergii* 6.7%, and *N. ionah* 6.0%). Interestingly, for prey species with a 1995–2014 habitat overlap < 10%, the viable habitat overlap is projected to increase for most species (by up to 7.2% for *L. aratrirostris* ; area to the left of the vertical dotted gray line in Figure 6a).

**FIGURE 7:**
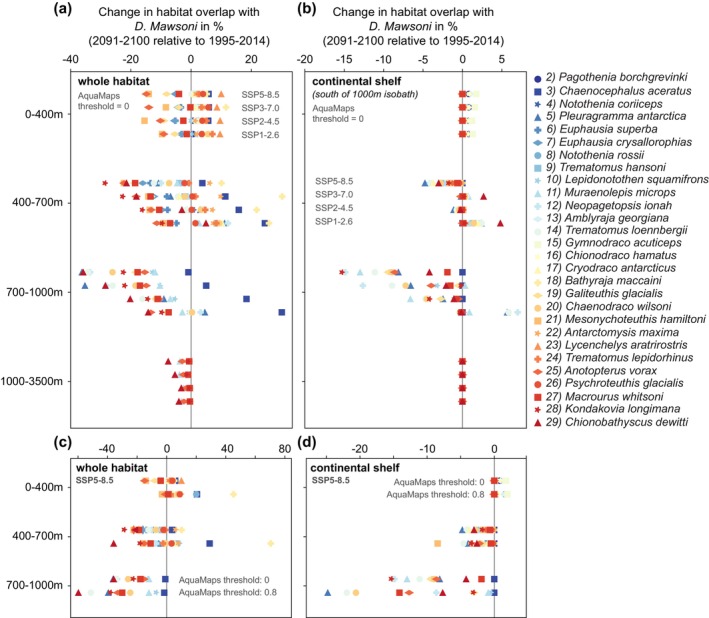
Drift‐corrected change in the viable habitat overlap (Φ; Equation 3) in % between 2091–2100 and 1995–2014 between the Antarctic toothfish ( *Dissostichus mawsoni* ) and its prey (a) in the whole *D. mawsoni* habitat and (b) in the fraction of the *D. mawsoni* habitat on the Antarctic continental shelf south of the 1000 m isobath (see Figure 1). Each prey species is denoted by a unique color‐symbol combination (see legend); the sorting of prey species corresponds to the sorting in Figure 2. The change in overlap is shown for the depth intervals 0–400 m, 400–700 m, 700–1000 m, and 1000–3500 m; the different rows within each depth interval correspond to the different emission scenarios. Panels (c) and (d) compare the results for the highest emission scenario SSP5‐8.5 using a species occurrence threshold of 0 and 0.8 (see Section 2.4). Note the different *x* axis scales compared to panels (a) and (b).

These changes in viable habitat overlap (Φ; Equation 3) are most pronounced at the subsurface (Figure 7), similar to the projected changes in environmental conditions (Section 3.1) and in viable habitat (Section 3.2). Changes in overlap are largest between 400 and 1000 m when considering the entire Antarctic toothfish habitat (Figure 7a), which is in line with the depth of strongest warming and deoxygenation (~500 m; see Figures 4, S6, S7). For these depth intervals, the decline in overlap amounts to more than 20% for the SSP5‐8.5 scenario between 400 and 700 m for Oceanic squid ( *K. longimana* ; 28.6%; red stars in Figure 7a), colossal squid ( *M. hamiltoni* ; 22.3%; orange squares), and Southern Ocean daggertooth ( *A. vorax* ; 22.3%; orange diamonds) and to more than 30% between 700 and 1000 m for Antarctic silverfish ( *P. antarctica* ; 36.3%; dark blue triangles), crocodile icefish ( *C. dewitti* ; 35.8%; red triangles), and Jonah's icefish ( *N. ionah* ; 33.5%; light blue crosses). Between 700 and 1000 m, the scenario uncertainty is especially large for some species, and the difference in viable habitat overlap decline between the highest emission scenario SSP5‐8.5 and the lowest emission scenario SSP1‐2.6 exceeds 40% for Antarctic silverfish ( *P. antarctica* ; dark blue triangles in Figure 7a) and 20% for crocodile icefish ( *C. dewitti* ; red triangles) and spiny icefish ( *C. wilsoni* ; orange circles). On the Antarctic continental shelf south of the 1000 m isobath (see black line in Figure 1), changes in overlap are generally smaller than for the entire Antarctic toothfish habitat (compare *x*‐axes in Figure 7a,b), and the decline in overlap amounts to up to 15% between 700–1000 m for the highest emission scenario for Oceanic squid ( *K. longimana* ) and Jonah's icefish ( *N. ionah* ; Figure 7b). Importantly, for the top 700 m of the water column, these findings are rather insensitive to the chosen probability threshold of species occurrence (illustrated for the SSP5‐8.5 scenario in Figure 7c,d). Between 700 and 1000 m, restricting the species habitats to regions of high occurrence probability (threshold of 0.8) increases the decline in projected habitat overlap with the Antarctic toothfish for some prey, for example, crocodile icefish ( *C. dewitti* ; from 35.8% to 59.8%; Figure 7c) and Antarctic silverfish ( *P. antarctica* ; from 8.1% to 24.8%; Figure 7d).

**FIGURE 8:**
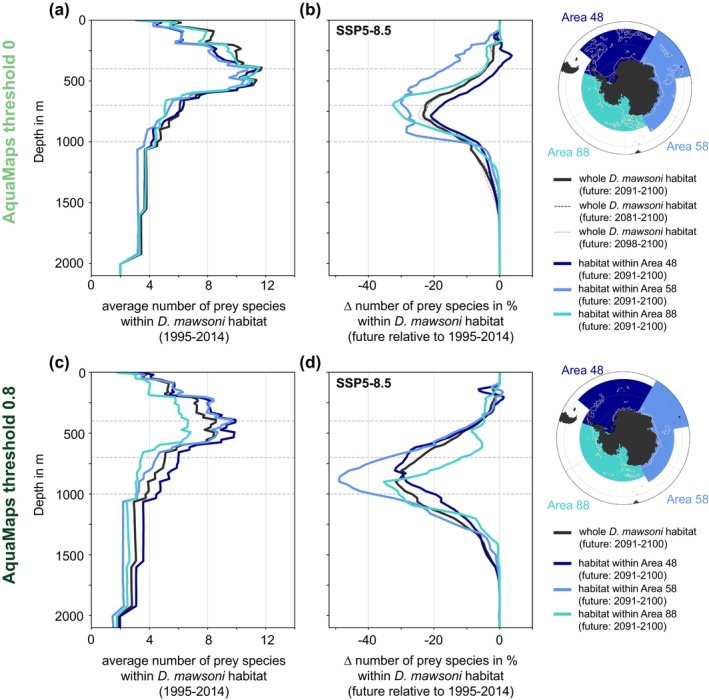
(a) Vertical profile of the AGI‐based average number of prey species available in the viable habitat of the Antarctic toothfish ( *Dissostichus mawsoni* ) for 1995–2014. The colors correspond to the whole *D. mawsoni* habitat (black) and the part of the habitat within the CCAMLR Convention Area 48 (Weddell Sea; dark blue), Convention Area 58 (East Antarctica; light blue), and Convention Area 88 (Ross Sea, Amundsen Sea, Bellingshausen Sea; mint) (CCAMLR Convention Areas 2017). See the map on the right for the location of the subareas. (b) Vertical profile of the change in the average number of prey species in % for 2091–2100 (SSP5‐8.5 scenario) relative to 1995–2014 within the *D. mawsoni* habitat (drift‐corrected). Dashed lines show the average prey number within the whole *D. mawsoni* habitat for 2081–2100 (dark gray) and 2098–2100 (light gray) relative to 1995–2014, respectively. Horizontal lines are shown at depths of 400 m, 700 m, and 1000 m, illustrating the depth intervals chosen for the results shown in Figures 6, 7, S1–S4. Panels (c) and (d) show the same as panels (a) and (b) but for a species occurrence threshold of 0 and 0.8 (see Section 2.4).

**FIGURE 9:**
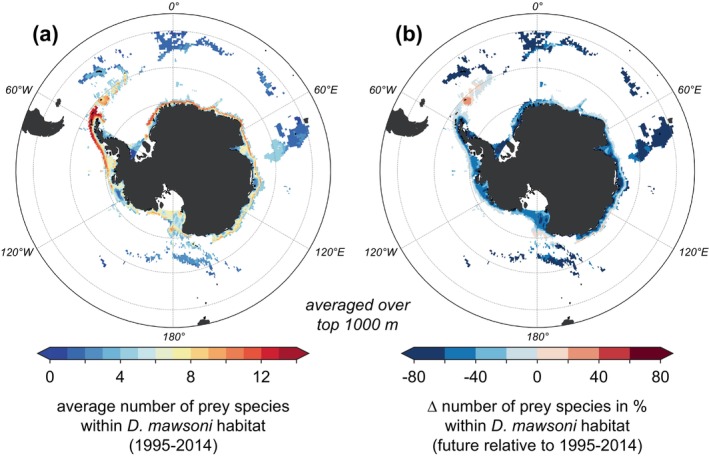
(a) Average number of prey species in the top 1000 m of the water column for 1995–2014 within the *D. mawsoni* habitat (drift‐corrected). (b) Change in % for 2091–2100 (SSP5‐8.5 scenario) relative to 1995–2014.

The species‐specific changes in viable habitat overlap with the Antarctic toothfish create hot spots of prey species loss around the Antarctic continent. Resulting from the quasi‐circumpolar distribution of most species (Figure 2), the area‐averaged number of available prey species 1995–2014 is similar throughout the water column for different sectors of the Southern Ocean, that is, the Weddell Sea and the Antarctic Peninsula (Area 48), East Antarctica (Area 58), and the Ross Sea, Bellingshausen Sea, and Amundsen Sea (Area 88; Figure 8) (CCAMLR Convention Areas 2017). For all regions, the number of prey species ranges from approximately four near the surface and below 1000 m to almost 12 at ~500 m depth (Figures 8a,c and 9a; based on each species' viable 3D habitat, see Figure 2), with numbers being overall lower if a higher threshold of species occurrence is used (compare Figure 8a,c). By the end of the 21st century, the number of available prey species within the Antarctic toothfish habitat decreases by up to ~30% for the highest emission scenario between 500 and 1000 m in Area 58 and Area 88, with the decline in Area 48 being lower (~20%; Figure 8b). Regionally, the change in the number of available prey can vary more drastically over the top 1000 m (Figure 9b). These numbers show little sensitivity to the chosen years for the future time slice (compare dashed gray lines to solid black line in Figure 8b), but the sensitivity is more pronounced for the chosen probability threshold for species occurrence (Section 2.4). Neglecting open‐ocean areas at lower latitudes in the calculation (threshold of 0.8) results in similar declines in prey availability in Areas 48 and 88, whereas the decline in Area 58 remains largest (up to 50%; Figure 8d). This analysis supports the existence of subsurface hotspots of prey loss in response to end‐of‐century warming and deoxygenation.

## 4 Discussion and Conclusions

Applying the AGI to output from 21st‐century model projections, our study demonstrates how changes in temperature and oxygen distributions alone could disrupt the interactions between the Antarctic toothfish, a top fish predator, and many of its prey. We find that the decline in viable habitat overlap is largest at the subsurface between 400 and 1000 m, which implies that Antarctic toothfish living at these depths are most susceptible to negative impacts. Indeed, while the highest catches of Antarctic toothfish are typically between 1000 and 1600 m, large catches (> 5 tons) have been recorded in depths as shallow as 500 m (Hanchet et al. 2015), which is consistent with the benthopelagic live history of the toothfish and its occurrence throughout the water column (Hanchet et al. 2008). Toothfish engage in vertical depth migrations due to buoyancy changes throughout their life history linked to lipid accumulation from their diet (Near et al. 2003). Adult toothfish have been hypothesized to experience cyclical buoyancy changes related to their condition level, with recently spawned fish in the northern parts of their distribution often in poorer condition and negatively buoyant after mobilizing protein and lipid stores for reproduction (Fenaughty, Eastman, and Sidell 2008). Thus, since prey availability is projected to be largely sustained in deeper waters (Figure 8), the most vulnerable individuals, which will likely be found at deeper depths (> 1000 m) due to their negative buoyancy and distribution off of the continental shelf, may be somewhat sheltered from the predicted shifts in prey distributions under climate change. Additionally, it remains unclear to what extent their opportunistic feeding behavior and thus a switch in dominant prey species could compensate for the projected regional loss of certain prey. Despite being opportunistic feeders (Hanchet et al. 2015), Antarctic toothfish have been suggested to be relatively sedentary (Grilly, Reid, and Thanassekos 2022), with only a small fraction (< 15%) of tagged Antarctic and Patagonian toothfish reporting at distances larger than 200 km from the release site (Grilly, Reid, and Thanassekos 2022; Kim et al. 2024). Acknowledging that exact travel pathways of tagged fish remained unresolved in existing studies (Grilly, Reid, and Thanassekos 2022; Kim et al. 2024), this suggests that, like related Antarctic fish species such as *Pleuragramma antarctica* (Hagen and Kattner 2017), toothfish engage in an energy‐saving life strategies that allows them to persist in the extreme conditions of the Southern Ocean (Caccavo et al. 2021). Any change in prey availability thus has the potential to reduce Antarctic toothfish abundance and distribution.

Based on our model projections of temperature and oxygen distributions, Antarctic toothfish populations in different sectors of the Southern Ocean would be affected in different ways by the decline in habitat overlap with its prey. For example, the Weddell Sea sector stands out as sustaining the highest number of prey by the end of the 21st century under the high‐emission scenario SSP5‐8.5 (> 80% are sustained relative to 1995–2014 at most depths; Figure 8b), with some depths above ~300 m even providing viable habitat for more prey species than during 1995–2014. Our analysis thus provides further support for the notion of the Weddell Sea as a climate change refuge, that is, an area with (i) a slower progression of environmental change than other sectors of the Southern Ocean (Nissen, Timmermann, et al. 2024) and (ii) comparatively small direct human impacts thus far from, for example, fishing or tourism (Teschke et al. 2021). We note that despite the sensitivity of the quantitative results in our study to the chosen probability threshold for species occurrence, the Weddell Sea remains among the regions with the highest number of sustained prey species even when a different threshold is used (Figure 8). In other sectors of the high‐latitude Southern Ocean, Antarctic toothfish populations are projected to be more severely affected than in the Weddell Sea (Figures 8b,d and 9b). Irrespective of the chosen probability threshold for species occurrence, our projections suggest highest prey loss in East Antarctica, where there has been a fishery for Antarctic toothfish since 2003, in conjunction with illegal, unreported and unregulated fishing (Yates et al. 2019). A similarly high prey loss is projected for the wider Ross Sea region when the open‐ocean habitat of Antarctic toothfish, which includes important spawning grounds (Hanchet et al. 2008; Parker et al. 2019), is included in the analysis (threshold of 0; Figure 8b). Thus, our results support the regional reduction or complete prohibition of toothfish fishing as part of the Ross Sea region Marine Protected Area to reduce the overall pressure on local ecosystems (Brooks et al. 2020). Further, to preserve the high‐latitude genetic, species and ecosystem diversity, our results suggest that the adoption of the proposed Weddell Sea and East Antarctic Marine Protected Areas should have high priority (Teschke et al. 2021; Brooks et al. 2020; Commission for the Conservation of Antarctic Marine Living Resources of Australia D. the European Union and its Member States 2016).

Even though Antarctic waters are not typically considered as oxygen‐poor (Breitburg et al. 2018), our results demonstrate the outsized role of changes in oxygen distributions over warming in driving future viable habitat changes of Antarctic species (Figures 4c, S4 and S5). The large change in oxygen distributions results from a redistribution of water masses, in particular, a southward shift of the relatively oxygen‐poor Circumpolar Deep Water at the subsurface (Nissen, Timmermann, et al. 2023; Nissen et al. 2022). Simultaneously, warming increases a species' oxygen demand (e.g., Pörtner and Peck (2011)), further aggravating the decline in viable habitat. Nonetheless, future oxygen levels remain well above the hypoxic levels which are threatening fish populations elsewhere (see, e.g., refs (Breitburg et al. 2018; Stramma et al. 2012)). That said, Antarctic species are specifically adapted to these comparatively high‐oxygen conditions, which is underscored by the higher ${\mathrm{pO}}_{2}$ thresholds for all species assessed here (${\mathrm{pO}}_{2}^{\mathrm{thr}}>$ 116 mbar in all 3D habitats; Table 1) than the globally distributed species assessed in Morée et al. (2023) (${\mathrm{pO}}_{2}^{\mathrm{thr}}>$ 35 mbar for all species). Acknowledging the potentially limited adaptation capacity of certain Antarctic ectotherms to adapt to deoxygenation due to the absence of hemoglobin in their blood (Ruud 1954; Kim et al. 2019), the results presented here illustrate how a decline in oxygen concentrations even at globally comparatively high reference oxygen levels could disrupt predator–prey interactions over the 21st century.

By definition, the AGI is designed to only assess contemporary habitat change, and it does not account for changes in key environmental factors beyond temperature and oxygen, which could further directly impact viable habitat overlap and predator–prey interactions. For example, many Antarctic species depend on sea‐ice cover as a spawning habitat (e.g., Antarctic toothfish/ *D. mawsoni* Parker et al. (2021); Behrens et al. (2024) and Antarctic silverfish/ *P. antarctica* Vacchi et al. (2012) or as a feeding ground for juveniles (Antarctic krill/ *E. superba* Meyer (2012 )). In response to recent changes in sea‐ice cover, the larval abundance of Antarctic silverfish has already declined near the Antarctic Peninsula Corso et al. (2022). Similarly, the life history of the Antarctic silverfish has been suggested to depend on regional circulation features, with flows onto the Antarctic continental shelf near, for example, the Filchner Trough in the Weddell Sea transporting adults toward spawning areas, while trough outflows transport developing juveniles toward the open ocean Caccavo et al. (2019). It can be expected that the substantial changes projected in Filchner Trough circulation, which include a reversal of predominant flow directions under the highest emission scenario SSP5‐8.5, will critically impact local silverfish populations and predator–prey interactions Nissen, Timmermann, et al. (2024). Furthermore, ocean acidification has been shown to increase the mortality of juvenile ploughfish/ *G. acuticeps* when acting in concert with warming and to reduce the metabolic capacity of, for example, black rockcod/ *N. coriiceps* and marbled rockcod/ *N. rossii* to adapt to warming (Flynn et al. 2015; Davis et al. 2018; Todgham and Mandic 2020). Acknowledging our incomplete understanding of ocean‐acidification impacts on many of the species assessed in this study (including their potential for adaptation), the severe levels of ocean acidification projected for the high‐latitude Southern Ocean under all but the lowest emission scenario SSP1‐2.6 could contribute to further disrupting food‐web integrity Nissen, Timmermann, et al. (2024). In summary, the inability of the AGI to (i) account for future extensions of the 3D contemporary habitat beyond the bounds prescribed here (Figures 1 and 2) and (ii) comprehensively include all key environmental variables defining a species' viable habitat implies that possible rearrangements of predator–prey interactions and ecosystem structure due to these shortcomings remain unconsidered in our analysis.

In conclusion, our results demonstrate how projected high‐latitude warming and deoxygenation alone can impact high‐latitude interactions between Antarctic toothfish and its prey under four 21st‐century emission scenarios. In the absence of directly accounting for additional key ecosystem drivers in our analysis based on the AGI (Clarke et al. 2021; Morée et al. 2023), such as ocean acidification and changes in sea‐ice cover or circulation, our findings highlight the need for the development of novel methodologies to quantify regionally resolved, cumulative climate change impacts on marine ecosystems under different future scenarios. Critically, for the development of robust methods, we need to improve our understanding of the life history of Antarctic species, of the connectivity of mobile fish populations between different sectors of the Southern Ocean, and of sensitivities of different species and life stages to changing environmental conditions. Our results clearly show the benefits for high‐latitude Southern Ocean organisms of following a lower emission pathway over the next decades, with changes in viable habitat overlap being reduced to < 20% for all prey species and depth levels for the lowest emission scenario SSP1‐2.6 (Figure 7). Yet, despite assuming our results to be qualitatively robust, the uncertainty associated with all quantitative findings of our study should be further assessed once more models of comparable model resolution and process representation become available. A more robust knowledge of projected high‐latitude climate change and subsequent ecosystem impacts is urgently needed to facilitate effective evaluation and sustainable management of Antarctic ecosystems (Brooks et al. 2020).

## Author Contributions

**Cara Nissen:** conceptualization, data curation, formal analysis, investigation, methodology, project administration, resources, software, validation, visualization, writing – original draft, writing – review and editing. **Jilda Alicia Caccavo:** data curation, investigation, methodology, writing – original draft, writing – review and editing. **Anne L. Morée:** data curation, investigation, methodology, writing – original draft, writing – review and editing.

## Conflicts of Interest

The authors declare no conflicts of interest.

## Supporting information


Data S1.

## Data Availability

All distribution data from AquaMaps were reviewed by Kathleen Kesner‐Reyes and shared with A. L. Morée between October 2022 and March 2023. A compilation of the species' two‐dimensional habitat masks regridded to the model grid can be accessed at Zenodo, see ref (Morée, Caccavo, and Nissen 2024) and ref.^?^. The calculated model fields of in situ temperature and *p*O_2_ are deposited at Zenodo, see ref (Nissen 2024). Original model fields can be accessed via ref (Nissen et al. 2023a) (*simA*, historical), ref (Nissen et al. 2023b) (*simA*, SSP1‐2.6), ref (Nissen et al. 2023c) (*simA*, SSP2‐4.5), ref (Nissen et al. 2023d) (*simA*, SSP3‐7.0), ref (Nissen et al. 2023e) (*simA*, SSP5‐8.5), and ref (Nissen et al. 2023f) (*simB*). The Fortran source code of FESOM1.4‐REcoM2 can be obtained via https://fesom.de/models/fesom14/ (last access January 23, 2023). The code version used for the simulations analyzed in this study is deposited at Zenodo, see ref (Nissen et al. 2023g) (Nissen, Moree and Caccavo 2024).
