# Supplementary material for: Twenty‐First‐Century Environmental Change Decreases Habitat Overlap of Antarctic Toothfish ( Dissostichus mawsoni ) and Its Prey
Source: Glob Chang Biol. 2025 Feb 10;31(2):e70063. doi: 10.1111/gcb.70063 (PMC11811694; doi:10.1111/gcb.70063)
Supplement: Supplementary file 1 — Data S1. [file GCB-31-e70063-s001.pdf]

21st-century environmental change decreases habitat overlap of Antarctic toothfish (*Dissostichus mawsoni*) and its prey

Cara Nissen [1,2,*] Jilda Alicia Caccavo [3,4] Anne L. Morée [5,6]

[1] *Department of Atmospheric and Oceanic Sciences and Institute of Arctic and Alpine Research, University of Colorado Boulder, Boulder, CO, USA.*
[2] *Now at: Department of Freshwater and Marine Ecology, Institute for Biodiversity and Ecosystem Dynamics, University of Amsterdam, Netherlands.*
[3] *Laboratoire des Sciences du Climat et de l'Environnement, LSCE/IPSL, CEA-CNRS-UVSQ, Université Paris-Saclay, Gif-sur-Yvette, France.*
[4] *Laboratoire d'Océanographie et du Climat Expérimentations et Approches Numériques, LOCEAN/IPSL, UPMC-CNRS-IRD-MNHN, Sorbonne Université, Paris, France.*
[5] *Climate and Environmental Physics, Physics Institute, University of Bern, Bern, 3012, Switzerland.*
[6] *Oeschger Centre for Climate Change Research, University of Bern, Bern, 3012, Switzerland.*
[*] Corresponding author: c.nissen@uva.nl

This supplementary material contains additional Figures and Tables in support of the main manuscript. The following Figures and Tables are included in this document:

Fig. S1: Maps of differences in pO_2 and in-situ temperature between WOA and FESOM-REcoM.

Fig. S2: Maps of historical pO_2 and its future change in FESOM-REcoM.

Fig. S3: Maps of historical in-situ temperature and its future change in FESOM-REcoM.

Fig. S4: Maps of AGI_{rel} in FESOM-REcoM.

Fig. S5: Vertical profiles of AGI_{rel} in FESOM-REcoM.

Fig. S6: Vertical profiles of future change in pO_2 in FESOM-REcoM.

Fig. S7: Vertical profiles of future change in in-situ temperature in FESOM-REcoM.

Table. S1: Overview of literature review on prey species of Antarctic toothfish.

Table. S2: Overview of literature review on prey species of Antarctic toothfish (continued).

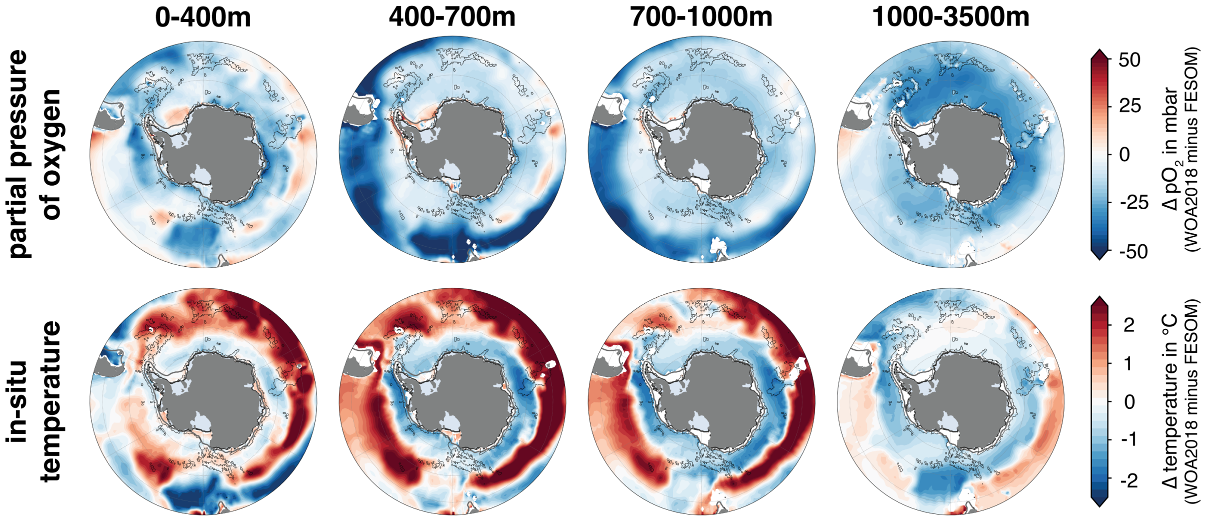

Figure S1: Maps of differences in partial pressure of oxygen in mbar and in-situ temperature in °C between World Ocean Atlas data and 1995-2014 FESOM-REcoM data for different depths. In all maps, the thin black line denotes the habitat of the Antarctic toothfish, and the thick black and grey line denote the ice-shelf front and the 1000 m isobath, respectively.

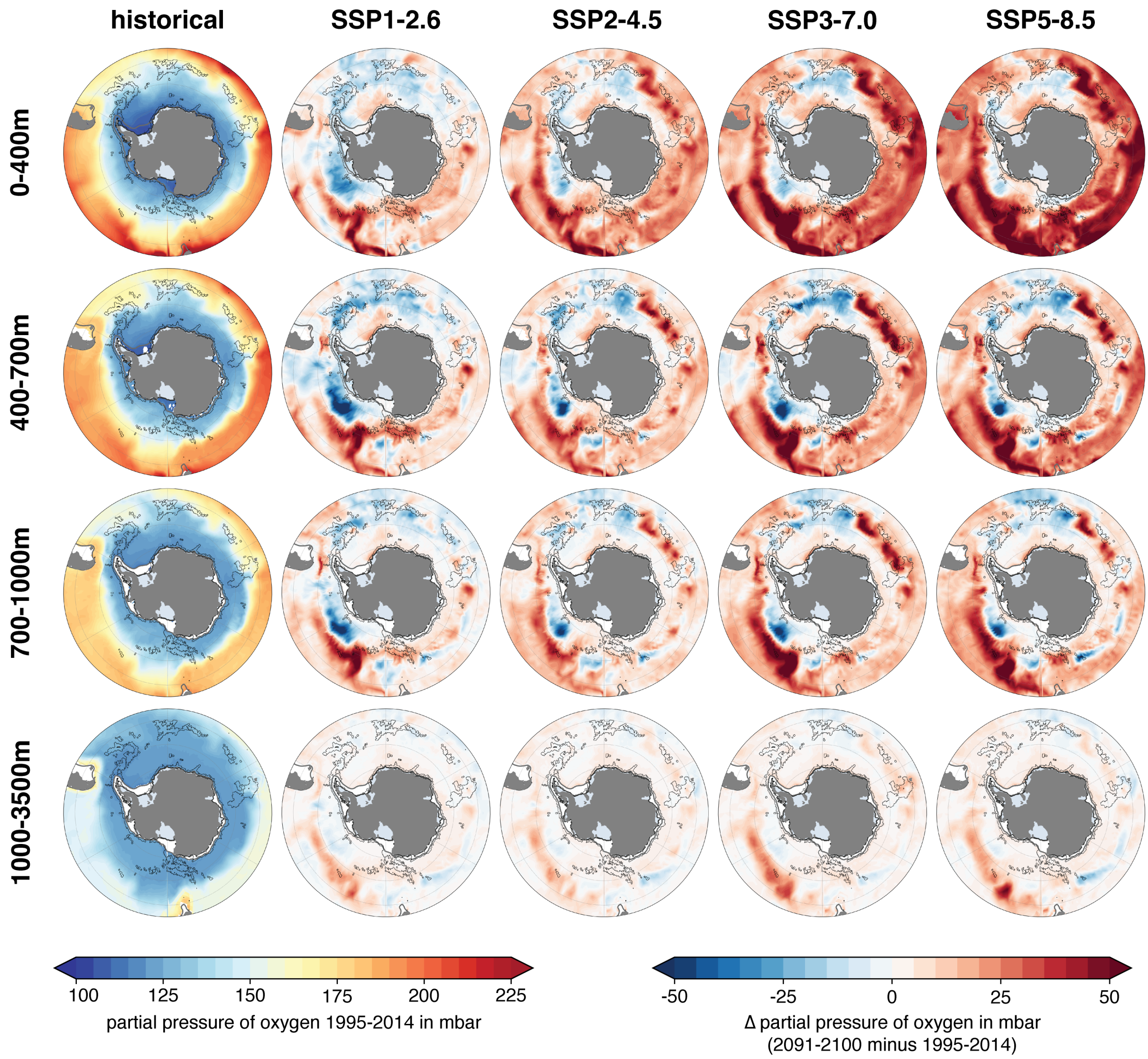

Figure S2: Maps of the partial pressure of oxygen in mbar for the historical period 1995-2014 and for the drift-corrected change between 2091-2100 and 1995-2014 for four emission scenarios and for 0-400 m, 400-700 m, 700-1000 m, and 1000-3500 m. In all maps, the thin black line denotes the habitat of the Antarctic toothfish, and the thick black and grey line denote the ice-shelf front and the 1000 m isobath, respectively.

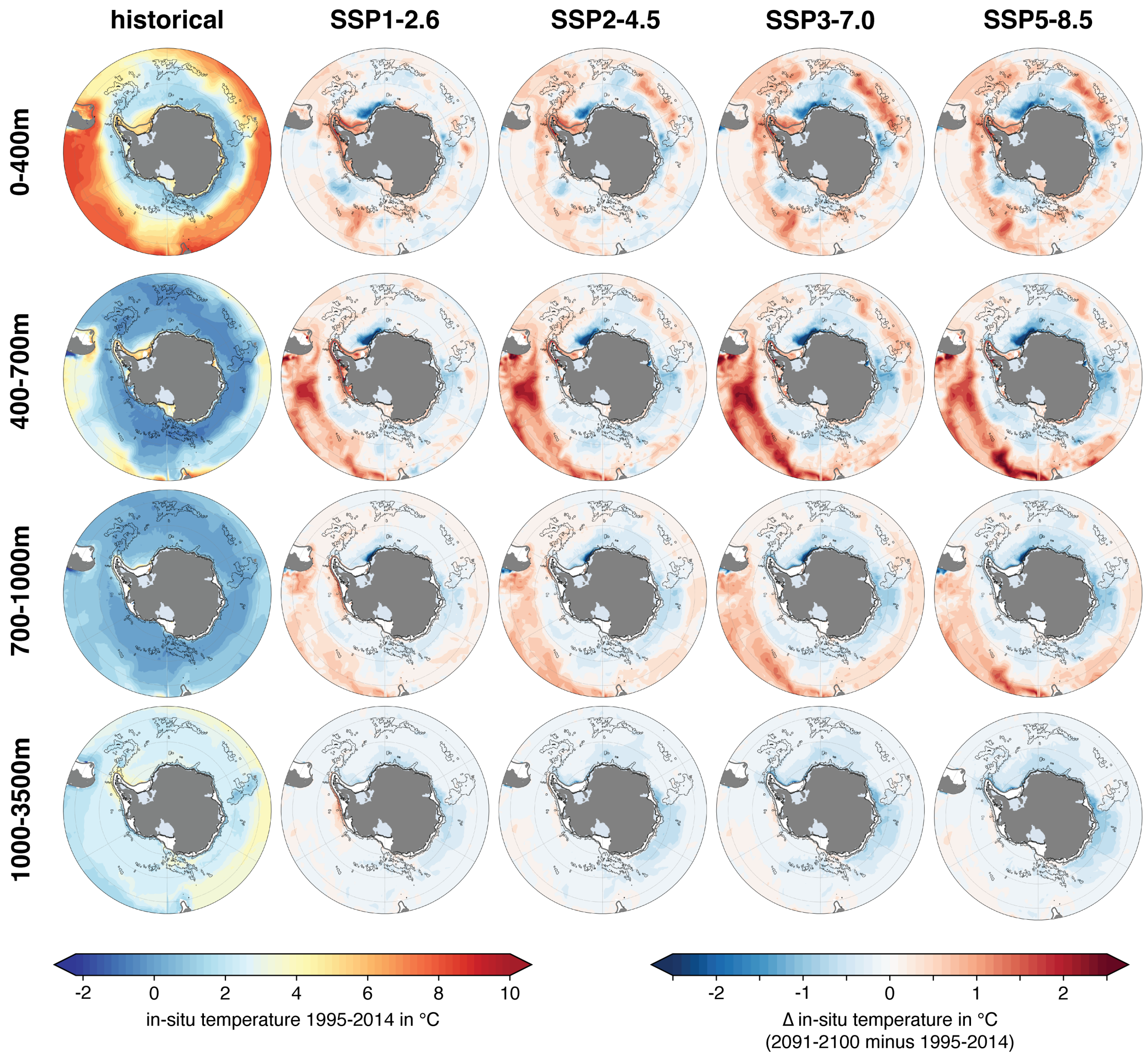

Figure S3: Maps of in-situ temperature in °C for the historical period 1995-2014 and for the drift-corrected change between 2091-2100 and 1995-2014 for four emission scenarios and for 0-400 m, 400-700 m, 700-1000 m, and 1000-3500 m. In all maps, the thin black line denotes the habitat of the Antarctic toothfish, and the thick black and grey line denote the ice-shelf front and the 1000 m isobath, respectively.

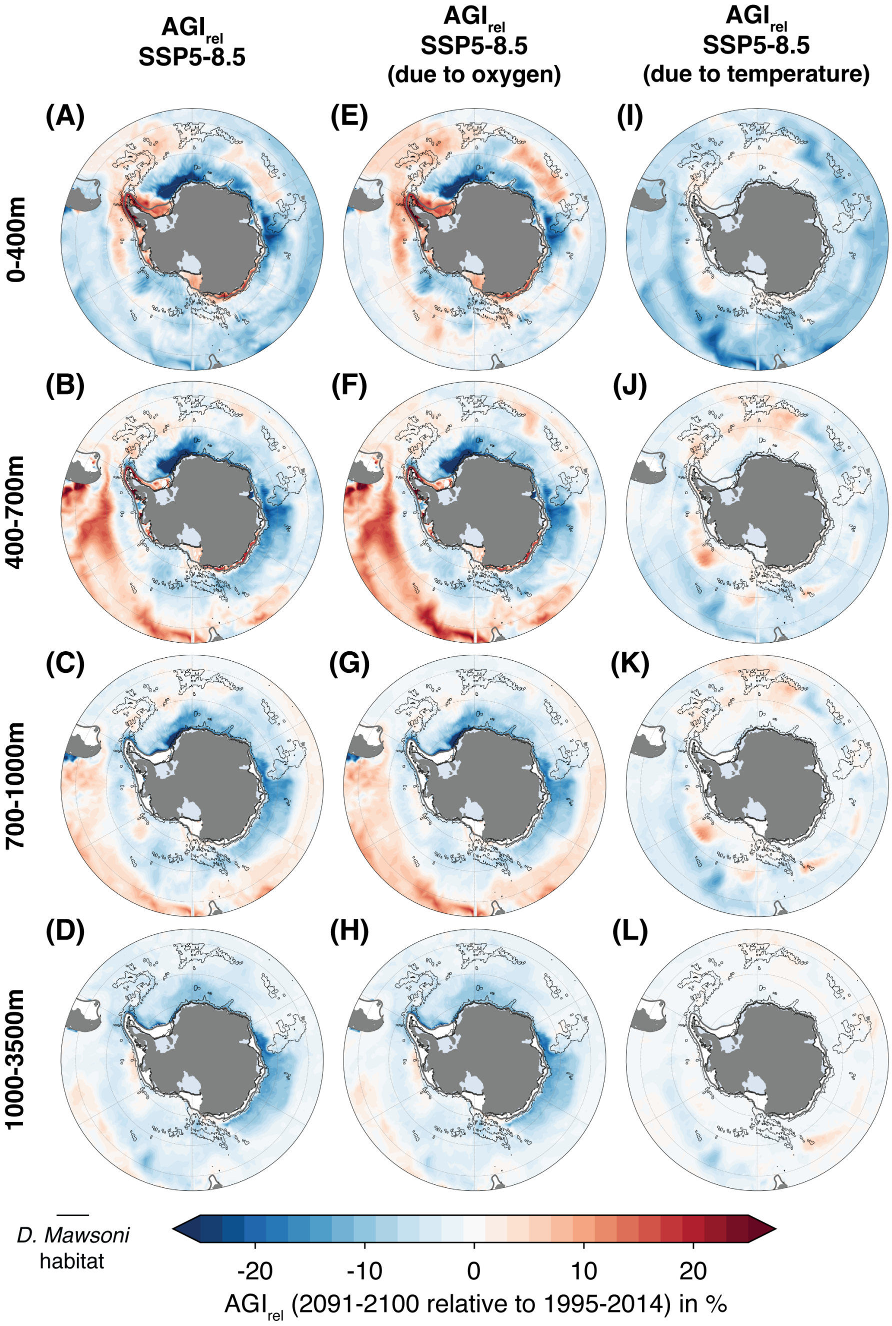

Figure S4: Relative change in the drift-corrected aerobic growth index (AGI$_{rel}$) in % between 2091-2100 for the SSP5-8.5 scenario and 1995-2014 for **(A)** 0-400 m, **(B)** 400-700 m, **(C)** 700-1000 m, and **(D)** 1000-3500 m. **(E)**-**(H)** Same as **(A)**-**(D)**, but only accounting for the future change in oxygen concentrations in the calculation of future pO_2 and AGI. **(I)**-**(L)** Same as **(A)**-**(D)**, but only accounting for the future change in in-situ temperature in the calculation of future AGI. In all maps, the thin black line denotes the habitat of the Antarctic toothfish, and the thick black and grey line denote the ice-shelf front and the 1000 m isobath, respectively.

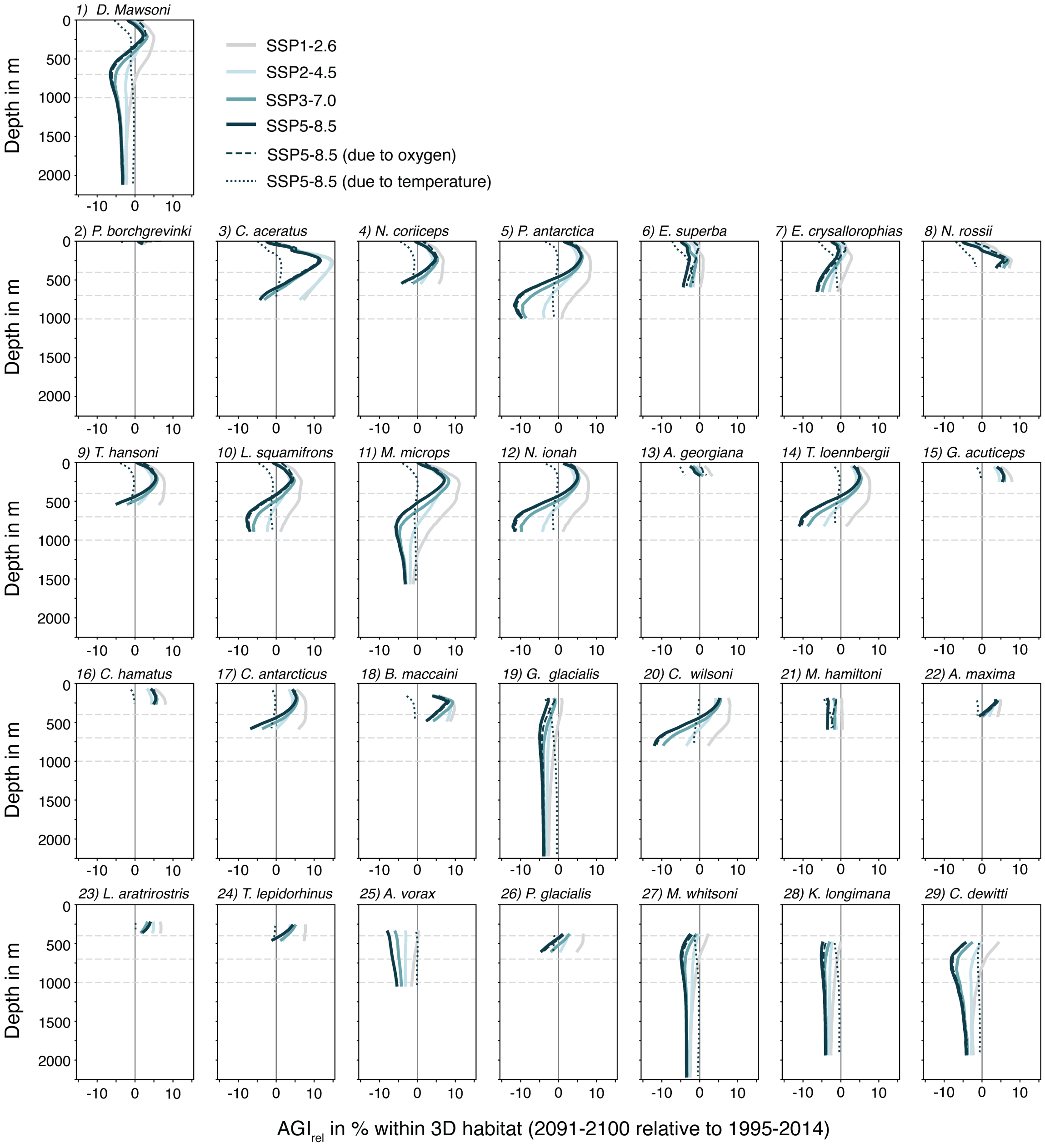

Figure S5: Vertical profiles of the drift-corrected relative change in the aerobic growth index (AGI$_{rel}$) in percent within the habitat of each species between 2091-2100 for the four emission scenarios and 1995-2014. In panel c), the dashed and dotted lines denote the future change due to only oxygen and temperature, respectively (SSP5-8.5 scenario). Horizontal lines are shown at depths of 400 m, 700 m, and 1000 m. The sorting of the species is identical to the sorting in Fig. 1 of the main text.

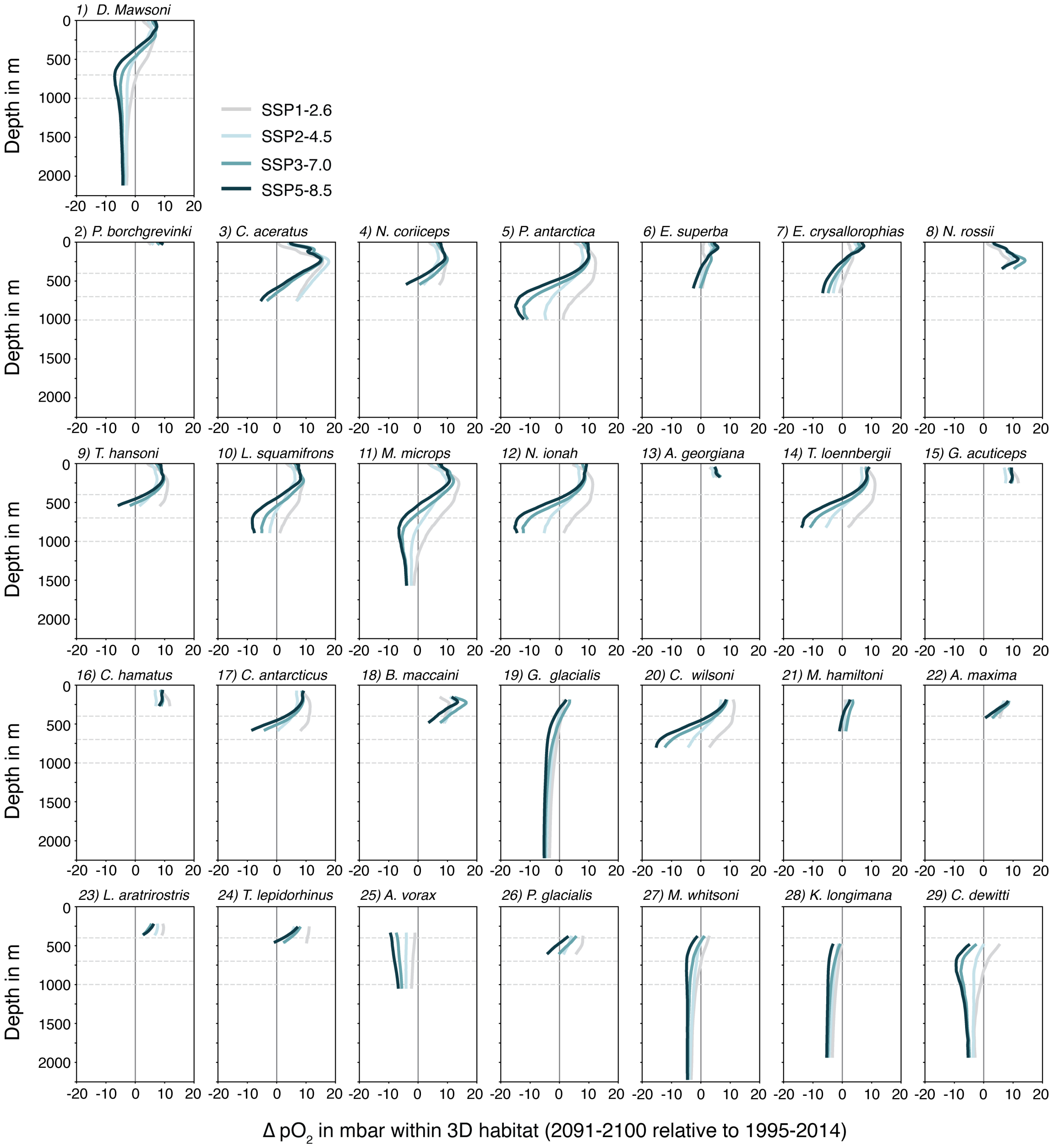

Figure S6: Vertical profiles of the projected change in partial pressure of oxygen (pO_2) in mbar within the habitat of each species between 2091-2100 for the four emission scenarios and 1995-2014. Horizontal lines are shown at depths of 400 m, 700 m, and 1000 m. The sorting of the species is identical to the sorting in Fig. 1 of the main text.

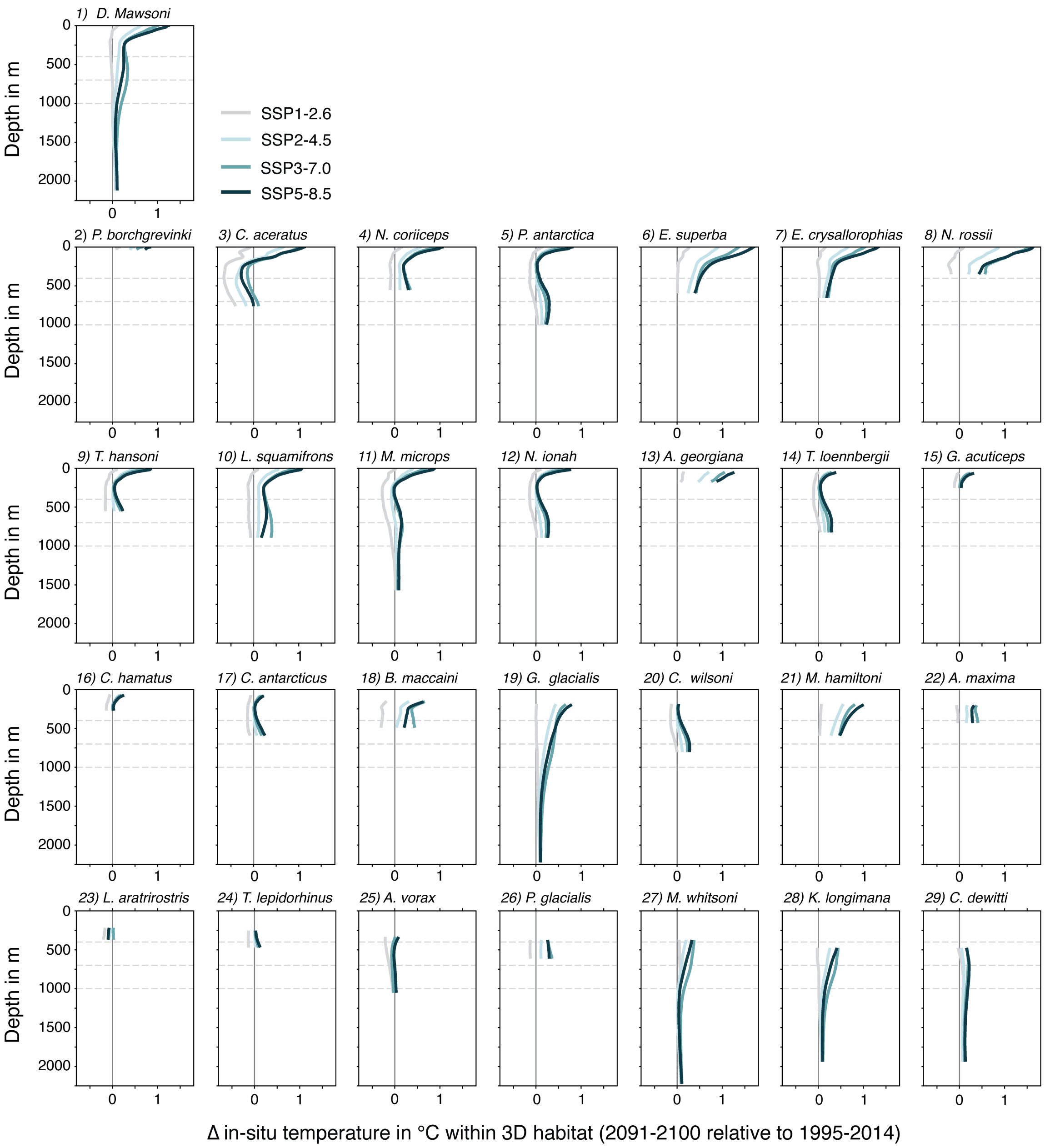

Figure S7: Vertical profiles of the projected change in in-situ temperature in °C within the habitat of each species between 2091-2100 for the four emission scenarios and 1995-2014. Horizontal lines are shown at depths of 400 m, 700 m, and 1000 m. The sorting of the species is identical to the sorting in Fig. 1 of the main text.

1 Supplementary Tables

Table S1: Overview of the literature review on prey species of Antarctic toothfish (*Dissostichus mawsoni*). The prey species included in the analysis are given in Table 1 of the main text. The reduced list was obtained by considering the abundance and frequency of prey species identified in the diet studies, as well as the extent to which prey species distributions were restricted to the Southern Ocean. Prey types are classified as F:=fish, C:=crustacean, S:=squid, SK:=skate, E:=echinoderm, O:=octopus, A:=arthropod, N:=nematode, and B:=bird. "n.a." is given when no information was available in the cited reference(s).

Refs.	**prey type**	**prey grouping**	**prey family**	**prey species**	**prey common name**
a,e,h,m	F	Superclass: Osteichthyes	Nototheniidae	*Pleuragramma antarctica*	Antarctic silverfish
a,m	F	Superclass: Osteichthyes	Nototheniidae	*Pagothenia borchgrevinki*	bald notothen
a,m	F	Superclass: Osteichthyes	Channichthyidae	*Pagetopsis macropterus*	n.a.
a,h,l,m	F	Superclass: Osteichthyes	Nototheniidae	*Trematomus loennbergii*	scaly rockcod
a,m	F	Superclass: Osteichthyes	Liparidae	*Paraliparis devriesi*	snailfish
a,i,m	F	Superclass: Osteichthyes	Bathydraconidae	*Gymnodraco acuticeps*	ploughfish
b,d-g,i-m	F	Superclass: Osteichthyes	Macrouridae	*Macrourus* spp.	grenadier
b,f,g,m	F	Superclass: Osteichthyes	Muraenolepididae	*Muraenolepis microps*	eel cod
b,d,f,g,j,l,m	F	Superclass: Osteichthyes	Moridae	*Antimora rostrata*	deepsea cod
b,m	F	Superclass: Osteichthyes	Artedidraconidae	*Pogonophryne permitini*	plunderfish
d-f,j-m	F	Superclass: Osteichthyes	Channichthyidae	*Chionobathyscus dewitti*	n.a.
d,m	F	Superclass: Osteichthyes	Channichthyidae	*Chionodraco hamatus*	n.a.
d,m	F	Superclass: Osteichthyes	Channichthyidae	*Cryodraco antarcticus*	long-fingered icefish
d,k,m	F	Superclass: Osteichthyes	Channichthyidae	*Neopagetopsis ionah*	Jonah's icefish
d,m	F	Superclass: Osteichthyes	Bathylagidae	*Bathylagus* spp.	deepsea smelt
d,f,k,l,m	F	Superclass: Osteichthyes	Anotopteridae	*Anotopterus pharao*	daggertooth
e,m	F	Superclass: Osteichthyes	Macrouridae	*Cynomacrurus piriei*	dogtooth grenadier
f,l,m	F	Superclass: Osteichthyes	Bathydraconidae	*Bathydraco marri*	dragonfish
f,g,k,m	F	Superclass: Osteichthyes	Nototheniidae	*Lepidonotothen squamifrons*	grey rockcod
g,m	F	Superclass: Osteichthyes	Nototheniidae	*Notothenia* spp.	rockcod
g,m	F	Superclass: Osteichthyes	Channichthyidae	*Chaenocephalus aceratus*	blackfin icefish
g,m	F	Superclass: Osteichthyes	Anotopteridae	*Anotopterus vorax*	daggertooth
g,m	F	Superclass: Osteichthyes	Bathydraconidae	*Bathydraco joannae*	dragonfish
g,m	F	Superclass: Osteichthyes	Stomiidae	*Borostomias antarcticus*	straightline dragonfish
g,k,m	F	Superclass: Osteichthyes	Zoarcidae	*Lycenchelys* spp.	eel pout
h	F	Superclass: Osteichthyes	Artedidraconidae	*Pogonophryne barsukovi*	stubbeard plunderfish
h,m	F	Superclass: Osteichthyes	Channichthyidae	*Dacodraco hunteri*	n.a.
i,l,m	F	Superclass: Osteichthyes	Paralepididae	*Arctozenus risso*	ribbon barracudina
i,m	F	Superclass: Osteichthyes	Bathydraconidae	*Cygnodraco mawsoni*	Mawson's dragonfish
k,m	F	Superclass: Osteichthyes	Paralepididae	*Magnisudis prionosa*	barracudina
k,m	F	Superclass: Osteichthyes	Lampridae	*Lampris immaculatus*	southern moonfish
k	F	Superclass: Osteichthyes	Nototheniidae	*Trematomus lepidorhinus*	slender scalyhead
k	F	Superclass: Osteichthyes	Artedidraconidae	*Pogonophryne scotti*	saddleback plunderfish
l,m	F	Superclass: Osteichthyes	Channichthyidae	*Chaenodraco wilsoni*	spiny icefish
l,m	F	Superclass: Osteichthyes	Macrouridae	*Coryphaenoides* spp.	rattail
l,m	F	Superclass: Osteichthyes	Nototheniidae	*Trematomus hansoni*	striped rockcod
l,m	F	Superclass: Osteichthyes	Zoarcidae	*Pachycara brachycephalum*	shorthead eel pout
e,m	F	Superclass: Osteichthyes	Liparidae	n.a.	snailfish
a,m	C	(unranked) order: Mysidacea	n.a.	Antarctomysis spp.	n.a.
a,m	C	Order: Amphipoda	Tryphosidae	Orchomenella spp.	n.a.
b,m	C	Order: Decapod	n.a.	Nauticaris spp.	prawn
b,m	C	Order: Isopoda	n.a.	n.a.	isopod
c,e,m	C	Order: Euphausiacea	Euphausiidae	n.a.	krill
c,m	C	Order: Mysida	Mysidae	n.a.	opossum shrimp
d-g,m	C	Order: Decapoda	Nematocarcinidae	Nematocarcinus spp.	prawn
d,m	C	Order: Isopoda	Serolidae	Ceratoserolis trilobitoides	n.a.
d,m	C	Order: Decapoda	Lithodidae	Paralomis birsteini	king crab
d,m	C	Order: Amphipoda	Eurytheneidae	Eurythenes gryllus	large scavenging amphipod
g,m	C	Order: Decapoda	Nephropidae	Thymops birsteini	Patagonian lobsterette
g,m	C	Order: Decapoda	Lithodidae	Paralomis spinosissima	Antarctic stone crab

a [1]: stomach contents, Ross Sea; b [2]: stomach contents, Ross Sea; c [3]: stomach contents,Southern Scotia Arc; d [4]: stomach contents, Ross Sea; e [5]: stomach contents, Ross Sea; f [6]: stomach contents, Lazarev Sea; g [7]: stomach contents, South Sandwich Islands; h [8]: fatty acids, Ross Sea; i [9]: stomach contents & fatty acids, East Antarctica; j [10]: stomach contents, Weddell Sea; k [11]: metabarcoding, Amundsen Sea & East Antarctica; l [12]: stomach contents, East Antarctica; m SCAR Diet and Energetics database [13]

Table S2: Table S1 continued.

Refs.	**prey type**	**prey grouping**	**prey family**	**prey species**	**prey common name**
b,m	S	Class: Cephalopoda	Octopoteuthidae	*Octopoteuthis rugosa*	n.a.
d,e,g,j,k,m	S	Class: Cephalopoda	Psychroteuthidae	*Psychroteuthis glacialis*	glacial squid
d,f,g,m	S	Class: Cephalopoda	Cranchiidae	*Mesonychoteuthis hamiltoni*	colossal squid
g,m	S	Class: Cephalopoda	Onychoteuthidae	*Moroteuthis knipovitchi*	smooth hooked squid
g,m	S	Class: Cephalopoda	Neoteuthidae	*Alluroteuthis antarcticus*	Antarctic neosquid
g,m	S	Class: Cephalopoda	Cranchiidae	*Galiteuthis glacialis*	glass squid
k	S	Class: Cephalopoda	Enteroctopodidae	*Muusoctopus levis*	n.a.
b,g,m	SK	Class: Chondrichthyes	Rajidae	*Amblyraja georgiana*	Antarctic starry skate
i,k,m	SK	Class: Chondrichthyes	Arhynchobatidae	*Bathyraja maccaini*	McCain's skate
i,m	SK	Class: Chondrichthyes	Arhynchobatidae	*Bathyraja eatonii*	Eaton's skate
b,m	E	Phylum: Echinodermata	n.a.	n.a.	n.a.
l,m	E	Class: Ophiuroidea	n.a.	n.a.	brittle star
l,m	E	Class: Asteroidea	n.a.	n.a.	sea star
d,f,g,m	O	Class: Cephalopoda	Onychoteuthidae	*Kondakovia longimana*	longarm octopus squid
d,m	O	Class: Cephalopoda	Megaleledonidae	*Megaleledone setebos*	giant Antarctic octopus
f,m	O	Class: Cephalopoda	Octopodidae	*Grimpoteuthis antarcticus*	umbrella octopus
g,m	O	Class: Cephalopoda	Stauroteuthidae	*Stauroteuthis gilchristi*	small pelagic octopus
k,m	O	Class: Cephalopoda	Cirroctopodidae	*Cirroctopus* spp.	barracudina
k,m	O	Class: Cephalopoda	Octopodidae	*Benthoctopus* spp.	n.a.
k,m	O	Class: Cephalopoda	Octopodidae	*Graneledone antarctica*	n.a.
k,m	A	Phylum: Arthropoda	n.a.	n.a.	n.a.
k	N	Phylum: Nematoda	n.a.	n.a.	n.a.
m	B	Order: Sphenisciformes	Spheniscidae	*Aptenodytes forsteri*	Emperor penguin

References

[1] Eastman, J. T. "The Evolution of Neutrally Buoyant Notothenioid Fishes: Their Specializations and Potential Interactions in the Antarctic Marine Food Web". *Antarctic Nutrient Cycles and Food Webs*. Ed. by Walter R. Siegfried, Pat R. Condy, and Richard M. Laws. Berlin, Heidelberg: Springer Berlin Heidelberg, 1985, 430–436. ISBN: 978-3-642-82275-9. DOI: 10.1007/978-3-642-82275-9_60.

[2] Fenaughty, J. M., Stevens, D. W., and Hanchet, S. M. "Diet of the Antarctic toothfish (Dissostichus mawsoni) from the Ross Sea, Antarctica (Subarea 88.1)". *CCAMLR Science* **10** (2003), 113–123. URL: https://www.ccamlr.org/en/publications/science_journal/ccamlr-science-volume-10/ccamlr-science-volume-10113-123.

[3] Near, Thomas J. et al. "Ontogenetic shift in buoyancy and habitat in the Antarctic toothfish, *Dissostichus mawsoni* (Perciformes: Nototheniidae)". *Polar Biology* **26** (2003), 124–128. ISSN: 1432-2056. DOI: 10.1007/s00300-002-0459-7.

[4] Stevens, D. W. *Report of the working group on fish stock assessment: Stomach contents of the Antarctic toothfish (Dissostichus mawsoni) from the western Ross Sea, Antarctica*. Report. 2004. URL: https://meetings.ccamlr.org/en/wg-fsa-04/31.

[5] Stevens, D. W. *Report of the working group on fish stock assessment: Stomach contents of sub-adult Antarctic toothfish (Dissostichus mawsoni) from the western Ross Sea, Antarctica*. Report. 2006. URL: https://meetings.ccamlr.org/en/wg-fsa-06/27.

[6] Petrov, A. F. and Tatarnikov, V. A. "Results of investigation of the diet of antarctic toothfish *Dissostichus mawsoni* (Nototheniidae) in the Lazarev Sea". *Journal of Ichthyology* **51** (2011), 131–135. DOI: 10.1134/S0032945210051017.

[7] Roberts, J, Xavier, Jose C, and Agnew, DJ. "The diet of toothfish species *Dissostichus eleginoides* and *Dissostichus mawsoni* with overlapping distributions". *Journal of Fish Biology* **79** (2011), 138–154. ISSN: 0022-1112. DOI: 10.1111/j.1095-8649.2011.03005.x.

[8] Jo, H.-S. et al. "Fatty acid and stable isotope analyses to infer diet of Antarctic toothfish caught in the southern Ross Sea". *CCAMLR Science* **20** (2013), 21–36. URL: https://www.ccamlr.org/en/publications/science_journal/ccamlr-science-volume-20/21\%E2\%80\%9336.

[9] Park, HJ et al. "Diet study of Antarctic toothfish caught in the east Antarctic based on stomach content, fatty acid and stable isotope analyses". *CCAMLR Science* **22** (2015), 29–44. ISSN: 1023-4063. URL: https://www.ccamlr.org/en/publications/science_journal/ccamlr-science-volume-22/29-44.

[10] Petrov, A.F. and Gordeev, I.I. "Distribution and biological characteristics of Antarctic toothfish *Dissostichus mawsoni* in the Weddell Sea". *Journal of Ichthyology* **55** (2015), 210–216. DOI: 10.1134/S0032945215020137.

[11] Yoon, Tae-Ho et al. "Metabarcoding analysis of the stomach contents of the Antarctic Toothfish (Dissostichus mawsoni) collected in the Antarctic Ocean". *PeerJ* **5** (2017), e3977. ISSN: 2167-8359. DOI: 10.7717/peerj.3977.

[12] Seong, Gi Chang et al. "Morphological dietary composition of Antarctic toothfish (Dissostichus mawsoni) along the East Antarctic continental slope". *Polar Biology* **44** (2021), 499–508. ISSN: 1432-2056. DOI: 10.1007/s00300-021-02820-9.

[13] Scientific Committee on Antarctic Research. *SCAR Southern Ocean Diet and Energetics Database*. 2023. DOI: 10.5281/zenodo.7796465.
